# Research Progress on Ultrahigh-Temperature Ceramics Modified C/C Composites

**DOI:** 10.3390/ma18163891

**Published:** 2025-08-20

**Authors:** Ruize Gao, Shuibin Wang, Tianci Zhou, Tianxing Jiang, Li Lu, Qingbo Wen, Shasha Tao, Xiang Xiong

**Affiliations:** 1 State Key Laboratory of Powder Metallurgy, Central South University, Changsha 410083, China; gao-ruize@csu.edu.cn (R.G.); 233311055@csu.edu.cn (T.Z.); xiongx@csu.edu.cn (X.X.); 2 School of Materials Science and Engineering, Central South University, Changsha 410083, China

**Keywords:** C/C composites, ultrahigh-temperature ceramics, oxidation resistance, ablation resistance, CMCs

## Abstract

C/C composites have excellent high-temperature mechanical properties and are one of the most promising high-temperature structural materials. However, poor oxidation and ablation resistance of the C/C composites in high-temperature oxygen-containing environments seriously hinder their practical applications. Ultrahigh-temperature ceramics (UHTCs)-modified C/C composites (C/C-UHTCs) are considered to be the most effective way to improve the oxidation and ablation resistance of C/C composites, demonstrating significant application prospects in aerospace and related fields. This article reviews recent research progress on C/C-UHTCs composites, including application prospects, performance testing methods, preparation techniques, phase composition, structural design principles, microstructural characteristics, as well as oxidation/ablation mechanisms. Addressing current challenges in C/C-UHTCs composites and future research directions are also proposed.

## 1. Introduction

**Figure 1:**
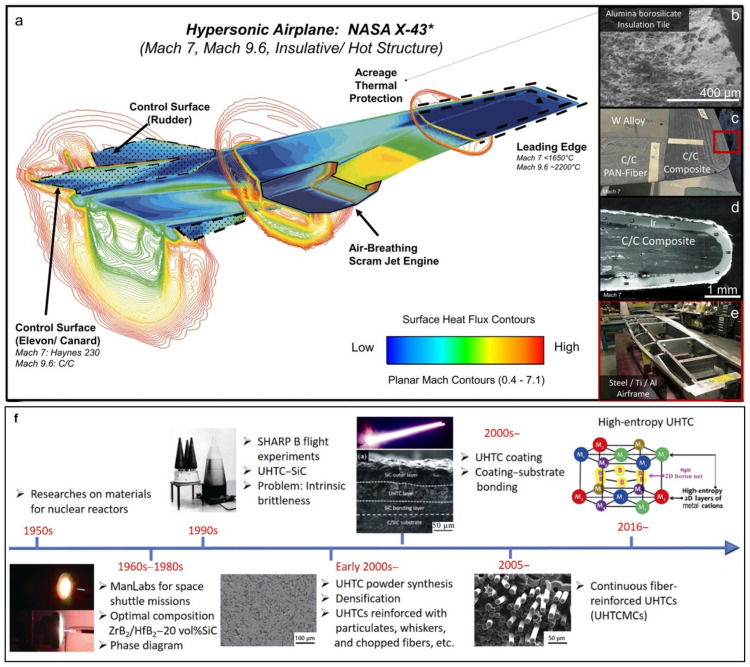
Computational fluid dynamics (CFDs) simulation, structural components, and the associated materials used for the X−43 hypersonic vehicle as well as historical perspective on research related to ultrahigh-temperature ceramics and composites: (**a**) CFD simulation results at a Mach 7 test condition with the engine operating; (**b**) aluminoborosilicate insulation tile with an emissive coating used for acreage protection thermal protection; (**c**) nose and leading−edge design integrating C/C composites and refractory tungsten alloy SD 180; (**d**) sharp leading edge cross-section showing the C/C composite with a refractory Ir coating; (**e**) airframe of the vehicle composed of steel/aluminum skin and Al/Ti bulkheads; and (**f**) historical perspective from 1950s to present. Copied with permission from Springer Nature [2,5].

With the rapid development of hypersonic vehicles, advanced aerospace facilities such as high-speed ballistic missiles, atmospheric re-entry vehicles, and hypersonic cruise aircraft have been successfully developed and tested, with top speeds reaching Mach 10 or even over Mach 20 [1]. Although the first hypersonic flight was developed around 70 years ago, there has been increasing interest from a broader audience due to the fact that modern engineering advances are poised to revolutionize suborbital travel, rapid access to space, and national defensive capabilities [2]. However, when vehicle speeds increase into the hypersonic regime, the physics of external aerodynamic flows become dominated by aerothermal heating (Figure 1a). Aerodynamic compression and friction create high-enthalpy gas dynamics that impart additional physical phenomena, including high heat fluxes (>hundreds of W/m), extreme thermal gradients (changing from −170 °C to 3000 °C across distances of order 1 cm), high stagnation pressures (~10^5^–10^7^ Pascals), as well as destructive plasma from gas ionization. With the increase in Mach numbers, these formidable phenomena must be accommodated by materials in the principal subsystems of hypersonic vehicles, such as aeroshell/primary structure, leading edges, control surfaces, acreage thermal protection, propulsion, and guidance systems (Figure 1b–e). Refractory metals, such as Wu, Mo, and Ti, have high melting points (Wu: 3422 °C, Mo: 2623 °C, and Ti: 1668 °C) that may meet the requirements (Figure 1c,e) [3,4]. However, these metals have rather high density and poor resistance to extreme environments. Therefore, there is an urgent need for lighter, stronger, and more resistant materials to replace them.

**Figure 2:**
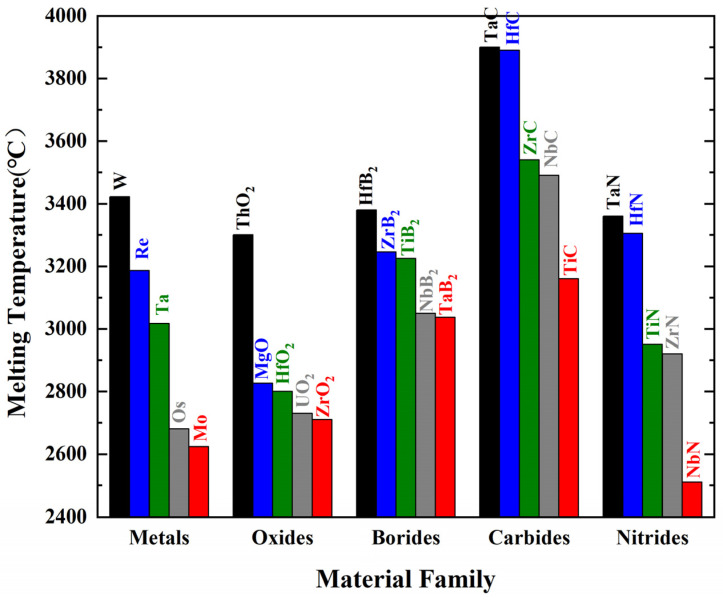
A comparison of the melting temperatures of the most refractory members of several classes of materials. Copied with permission from John Wiley and Sons [7]. The borides, carbides, and nitrides with the melting points higher than 3000 °C are considered as ultrahigh-temperature ceramics.

Ultrahigh-temperature ceramics (UHTCs) refer to thermal structural ceramics with melting points above 3000 °C that can stably exist in high-temperature, oxygen-rich environments exceeding 2000 °C [6]. These typically include carbides, borides, oxides, and nitrides of refractory metals (Figure 2) and their multicomponent composite ceramics [7]. Table 1 lists the physical and mechanical properties of several boride, carbide, and nitride-based UHTCs [5]. It is evident that UHTCs have extremely high melting points (>3000 °C), high thermal conductivity, and very high strength and hardness, making them highly promising materials for ultrahigh-temperature thermal protection [8]. Figure 1f shows the historical perspective on research related to ultrahigh-temperature ceramics and composites [5]. The earliest research on UHTCs can be traced back to the 1950s, when HfB_2_ and ZrB_2_ were used as nuclear reactor materials because of their excellent high-temperature corrosion resistance [9]. Starting from the 1960s, the UHTCs were considered as thermal protection materials by the ManLabs laboratory funded by the Air Force Materials Laboratory of the USA [5,10]. At the end of the 1990s, the demonstration tests of the thermal protection structure of UHTCs carried out by Ames and Glenn Research Center together with Sandia National Laboratory and the Air Force Space Command proved their great potential in extreme environments from the viewpoint of ablation resistance, although they exposed the intrinsic brittleness [7]. Therefore, strengthening and toughening the UHTCs have become research hotspots in recent years. The fracture toughness of bulk UHTCs can be improved to a certain extent by introducing particulates, whiskers, or platelets as reinforcements. However, the intrinsic brittleness cannot be eliminated completely. In addition, due to ultrahigh processing temperatures of bulk UHTCs, it is difficult to prepare components with complex shapes on a large scale. So, it is challenging to directly apply them in thermal protection systems (Figure 1f) [5,11]. Starting from ~2005, a large number of studies regarding continuous fiber-reinforced ultrahigh-temperature ceramic matrix composites (UHTCMCs) have demonstrated that introducing continuous fibers into the matrix is a better solution to toughen the UHTCs and overcome their inherent brittleness and poor thermal shock resistance [12,13]. Since then, UHTCMCs have attracted much attention, and the number of relevant publications has increased rapidly every year [5]. Nevertheless, the density of the continuous fiber-reinforced ultrahigh-temperature ceramic matrix composites, as well as their high-temperature mechanical properties, still need to be optimized.

**Figure 3:**
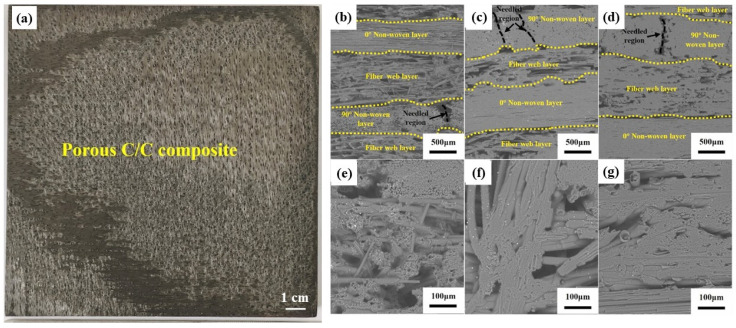
Photograph of a typical C/C composite (**a**) and SEM images of the cross-sections of C/C composites with different densities (**b**,**e**): 1.0 g/cm^3^; (**c**,**f**): 1.2 g/cm^3^; and (**d**,**g**): 1.4 g/cm^3^. Copied with permission from Elsevier [14].

C/C composites, namely carbon fiber reinforced carbon matrix composites, consist of carbon fibers (generally in the form of non-woven fabrics, fiber webs, and needle fiber bundles), PyC interface, carbon matrix, as well as minimal voids or defects. Figure 3 shows a photograph of a typical C/C composite and SEM images of the cross-sections of C/C composites with different densities [14]. As shown in Figure 3, the C/C composites with low density (1.0~1.2 g/cm^3^) have large pores between the fiber bundles and micro-sized pores inside the fiber bundles (Figure 3b,c,e,f). For the C/C composites with higher density (≥1.4 g/cm^3^), most of the pores are located between the fiber bundles (Figure 3d,g). The properties of C/C composites are closely linked to their microstructure. Their mechanical properties, including tensile, flexural, and compressive strength, as well as thermal properties like thermal conductivity and coefficient of thermal expansion, can be tailored by adjusting parameters such as fiber orientation, volume fraction, yarn architecture, and manufacturing processes like carbonization temperatures and densification techniques. For instance, their thermal conductivity along the fiber axis can reach up to 1000 Wm^−1^ K^−1^, significantly higher than in the transverse direction [15].

**Figure 4:**
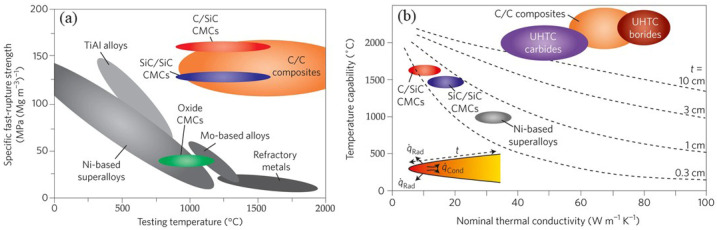
Comparison of high temperature properties of C/C composites and other materials. (**a**) Specific fast-rupture strength as a function of the temperature of various metals and composites. (**b**) An Ashby-type map of temperature capability and nominal thermal conductivity of different materials, including Ni-based superalloys, CMCs, UHTCs, and C/C composites. Copied with permission from Springer Nature [18].

As reported in the literature, C/C composites attracted much attention in the last decades because they boast low density, high specific strength, low thermal expansion coefficient, and superior thermal shock resistance [16,17]. As shown in Figure 4, their high-temperature performance far surpasses that of traditional metal-based materials, making them widely used in the thermal structural components of various aerospace vehicles (Figure 1c,d) [2,11,18,19,20,21]. Notably, C/C composites are the only materials whose mechanical properties do not degrade but rather improve at temperatures above 2000 °C, which is even better than that of the continuous fiber-reinforced ultrahigh-temperature ceramic matrix composites (UHTCMCs). However, C/C composites begin to oxidize and fail at temperatures above 500 °C, severely hindering their application in fields such as aerospace [17,22].

In recent years, UHTCs-modified C/C composites have been developed, which integrate the advantages of UHTCs with C/C composites and effectively resolve the aforementioned challenges, significantly advancing their application in thermal protection systems. This is because the UHTCs can enhance the ablation resistance of the C/C composites; meanwhile, the C/C composites provide excellent high-temperature strength, thermal shock resistance, and low density. In contrast to externally applied coating protection, matrix modification by UHTCs can enhance the oxidation and ablation resistance of C/C composites by altering their base composition. These modifications not only reduce oxygen contact with the matrix but also improve thermal conductivity. Most importantly, the surface of UHTCs-modified C/C can form a dense oxide layer or ceramic skeletons after oxidation, which can effectively block the diffusion of oxygen and the erosion caused by high-temperature heat flow [23]. In the last decade, extensive research has been conducted on UHTCs-modified C/C composites, yielding significant advancements. However, practical applications remain constrained by challenges such as low thermal conductivity and high thermal expansion coefficients of the oxides of UHTCs, which induce cracking and delamination. Therefore, this paper systematically reviews the recent research progress in UHTCs-modified C/C composites, including both achievements and persistent limitations, and aims to provide insights for further optimizing the thermal protection performance of these advanced materials.

## 2. Preparation Methods and Microstructure of the UHTCs-Modified C/C Composites

After years of development of C/C composites and the maturity of the preparation process of UHTCs-modified C/C composites, a comprehensive manufacturing system for these materials has been established. The main processes, their features are summarized as follows.

### 2.1. Chemical Vapor Infiltration

**Figure 5:**
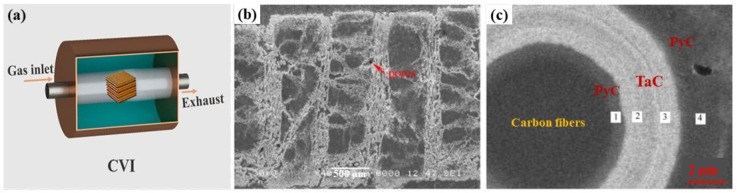
(**a**) Schematic diagram of chemical vapor infiltration (CVI) and (**b**,**c**) microstructure of C_f_/C-TaC composites prepared using CVI. Copied with permission from American Chemical Society and Elsevier [26,27].

Chemical vapor infiltration (CVI) originated from chemical vapor deposition (CVD). This process involves infiltrating reaction gas (precursors) into the interior through the pores on the surface of the substrate, followed by thermal decomposition to form a ceramic matrix. Figure 5 shows the schematic diagram of CVI and the microstructure of C/C-TaC composites prepared using CVI, possessing a unique matrix with a PyC/C-TaC/PyC layered-structure. The common reaction equations for preparing HfC, ZrB_2_, and TaC ceramic matrix by CVI are as follows [24,25]:

(1) $${HfCl}_{4}+{CH}_{4}+H_{2}\overset{Ar}{\to }HfC+4HCl$$

(2) $${ZrCl}_{4}+2{BCl}_{3}+5H_{2}\overset{Ar}{\to }{ZrB}_{2}+10HCl$$

(3) $${6TaCl}_{5}+{2C}_{3}H_{6}+9H_{2}\overset{Ar}{\to }6TaC+30HCl$$

The main advantages of the CVI include the following: (1) low-temperature/pressure operation, resulting in low residual stress within the matrix, minimal damage to carbon fibers, and high material performance; (2) uniform gas flow distribution, enabling the production of complex-shaped components; and (3) controllable design flexibility, resulting in improved densification, compromising oxidation resistance and high-temperature mechanical properties. The primary limitations of the CVI process are as follows: (1) low efficiency and poor utilization of carbon sources/precursors leads to prolonged cycles and high costs; (2) surface pore blockage, causing density gradients during infiltration and hindering subsequent densification; and (3) structural inhomogeneity, leading to reduced densification, compromising oxidation resistance and high-temperature mechanical properties [28,29,30].

### 2.2. Precursor Infiltration and Pyrolysis

**Figure 6:**
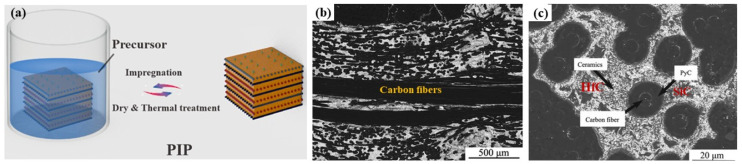
(**a**) Schematic diagram of PIP and (**b**,**c**) microstructure of C/C-SiC-HfC composites prepared via PIP. Copied with permission from American Chemical Society and Springer Nature [26,32].

The precursor infiltration and pyrolysis (PIP) method mainly consists of the following processes: (1) synthesis of metal-containing liquid preceramic polymers; (2) infiltration into the preforms under pressure; and (3) formation of ceramic matrix through crosslinking and pyrolysis at appropriate temperatures [25,31]. Generally, because the shrinkage of precursors leads to pores and cracks after pyrolysis, the PIP process must be repeated severally times. Figure 6 shows the schematic diagram of the PIP process and microstructure of C/C-SiC-HfC composites prepared via precursor infiltration and pyrolysis [32].

The advantages of the PIP process are as follows: (1) tailorable precursors: The precursor has excellent designability, allowing for optimized bonding between fibers and the matrix; (2) low-temperature processing: The pyrolysis temperature is relatively low (≤1500 °C), making it less likely to damage carbon fibers; and (3) deep infiltration capability: The precursor can deeply penetrate the preform, enabling the production of complex-shaped components. Nevertheless, high-temperature decomposition of the precursors with gaseous molecules escaping during the PIP process leads to high open porosities in the matrix. It requires multiple cycles, resulting in a longer preparation time and higher production cost (the cost of precursors of UHTCs is rather high). In addition, pyrolysis can cause volume shrinkage, generating micro-cracks and stress within the matrix.

### 2.3. Reactive Melting Infiltration

**Figure 7:**
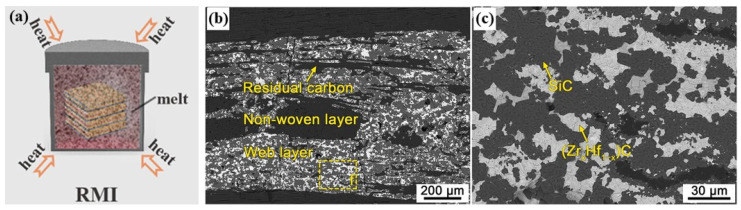
(**a**) Schematic diagram of reactive melting infiltration and (**b**,**c**) microstructure of C/C-SiC-(Zr_x_Hf_1−x_)C composites prepared via RMI process. Copied with permission from American Chemical Society and Elsevier [26,33].

The reactive melting infiltration (RMI) method involves encapsulating C/C preforms with metal powders and heating them to above the melting point of the metals in a vacuum (Figure 7). The molten metal then penetrates the porous substrate under capillary action and rapidly reacts with C or B elements to form a UHTC matrix. The advantages of the RMI method include the following: (1) short preparation time; (2) low manufacturing cost; (3) ease of achieving near-net shape, high material density, and superior performance; and (4) additionally, it can also produce parts with complex geometries.

According to infiltration dynamics, molten metal penetration is influenced by viscosity, surface tension, wetting angle θ, and substrate porosity [34,35]. The formation and growth of carbide layers depend on the diffusion of C atoms and metal atoms. Therefore, insufficient control over reaction progress and material usage during RMI can lead to incomplete reaction, leaving residual metals in the matrix. This causes residual stress and interface mismatch, reducing high-temperature creep resistance and ablation resistance. Additionally, excess molten metal reacting with carbon fibers can reduce the mechanical properties of the composites. Furthermore, the formation of UHTCs requires infiltration temperatures typically 100–200 °C above their melting points (Zr melts at 1855 °C and Hf at 2227 °C). This not only increases equipment burden but also involves high-temperature heat treatment that can cause graphitization of the pyrolytic carbon, further degrading the mechanical properties of the composites [36].

### 2.4. Combined Preparation Technology

As mentioned above, there are always some disadvantages in the preparation methods of the UHTCs-modified C/C composites, regardless of CVI, PIP, or RMI. The CVI method causes minimal damage to the carbon fibers but tends to produce pores, and it is time-consuming. The PIP process requires low temperatures, but it is prone to micro-cracks in materials and has lower efficiency. RMI is highly efficient but can damage fibers due to the high-temperature melts. Therefore, researchers often design flexible preparation processes by combining two or more methods to optimize material properties [14]. For example, Yao et al. [37] used a 2.5D needle-punched carbon fiber preform as the matrix, sequentially depositing PyC and SiC phases through the CVI process, introducing a ZrC-SiC binary phase via PIP, and finally depositing a SiC coating via CVD to prepare composites with different CVI-derived C-SiC volume ratios. In the CVI stage, PyC and SiC fill the pores sequentially, while in the PIP stage, ZrC-SiC fills the remaining large pores. As the C-SiC volume ratio increases, the PyC layer thickens, the SiC content decreases, the density of the composite material decreases, and the porosity rate reduces. Zhou et al. [38] prepared a C/C-ZrC-Cu material using the CVI + RMI method, using an integrated needle-punched carbon fiber felt as the preform. After chemical vapor infiltration densification, a Zr-40 wt% Cu mixed powder was used as the infiltration agent, and the material was formed at 1350 °C for 60 min. This combined the initial densification of CVI with the ZrC formation and densification of RMI. Erosion tests showed that chemical erosion predominated initially, with Cu melting/evaporation providing cooling; later, mechanical erosion took over. After ZrO_2_ melted, it filled cracks and voids, with the glassy ZrO_2_ layer enhancing both chemical and mechanical erosion resistance. Wen et al. [14] prepared C/C-ZiC-ZrC-SiC composites by a combination of PIP and RMI processes on porous C/C composites. The PIP is used for preparing a ZrC-SiC interlayer (denoted as ZiC) between the C/C composites and ZrC-SiC matrix prepared by RMI. The results show that the increase in both flexural and tensile strength of the composites after RMI can be optimized by adjusting the ZiC and achieve 50.54% and 56.76%, respectively. The mechanisms can be attributed to the facts that the ZiC effectively protects carbon fibers and PyC from serious erosion by high-temperature melt and weakens the interfacial bond between the ceramic matrix and carbon fibers, ensuring a series of stress release under external load.

**Figure 8:**
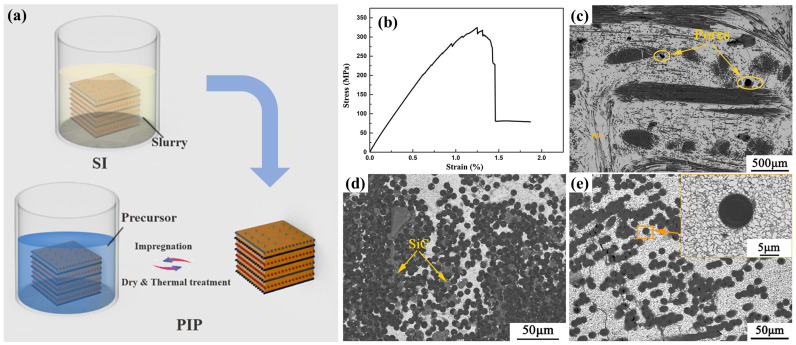
(**a**) Schematic diagram of SI combined with PIP, as well as (**b**) bending stress-strain curve and microstructure (**c**–**e**) of the C/C-HfB_2_-SiC composites prepared via SI + PIP process (inset in (**e**) is the enlarged image showing the cross section of one carbon fiber within the ceramic matrix). Copied with permission from American Chemical Society and Elsevier [26,44].

Regarding the low efficiency of the process mentioned above, pre-infiltration/loading of UHTC powders in the porous C/C composites or carbon fiber preforms before a continuous densification process (such as CVI, PIP, and RMI) is a promising strategy. Combined with this technique, the introduction of UHTCs into porous C/C composites or carbon fiber preforms becomes simple and efficient. The composition, quantity, and distribution of UHTC powders can be well controlled, providing more options for subsequent densification processes. One of the most cost-effective, simple, design flexible and rapid pre-infiltration/loading methods is slurry infiltration (SI), which involves forming a stable suspension by mixing ceramic powder with solvents and dispersants, then infiltrating it into fiber preforms or porous C/C composites using pressureless or pressure-assisted methods, followed by drying and debinding to remove solvents and organic components [25,39,40,41]. Zhou et al. prepared C/C-ZrC and C/SiC-ZrC composites employing a joint process of SI and CVI, in which the ZrC matrix was obtained by the SI process, while the C or SiC matrix was introduced by the CVI process. The tensile strength of the as-fabricated C/C-ZrC and C/SiC-ZrC composites is 89.4 ± 8.4 MPa and 182.2 ± 14.0 MPa, respectively. The linear ablation rates of the C/C-ZrC and C/SiC-ZrC composites under air plasma are 4.8 mm s^−1^ and 3.9 mm s^−1^, respectively [42]. Tang et al. prepared C/C-ZrC-SiC and C/C-HfB_2_-SiC composites using a high-solid-loading SI process combined with the PIP process (Figure 8a) [43,44]. For the C/C-ZrC-SiC composite, they found that when introducing around 0.4 wt% polyethyleneimine (PEI), the ZrC aqueous slurry exhibits the lowest viscosity (60.2 mPa·s) and the highest solid content (38 vol.%). After the combined densification process, the as-obtained C/C-ZrC-SiC composite shows a density of 2.74 g/cm^3^ and an open porosity of 8.57%. After ablation under an oxyacetylene flame with a heat flux of 3.86 MW/m^2^ for 120 s, the linear and mass ablation rates of the composites were as low as 0.72 ± 0.11 mg/s and 0.52 ± 0.09 µm/s, respectively [43]. The C/C-HfB_2_-SiC composite shows a pseudo-plastic fracture behavior with bending strength up to 344.8 MPa (Figure 8b). The microstructure characterization of the composites reveals that HfB_2_ particles are uniformly filled in the pores in the C/C preform (Figure 8c), and SiC well densifies the interstices between HfB_2_ particles and the small pores in the carbon fiber bundles (Figure 8d,e). After ablation with oxyacetylene flame at 2500 °C for 120 s, the mass ablation rate and linear ablation rate of the composite are 0.5 mg/s and 0.415 μm/s, respectively. The excellent ablation performance is attributed to the hindering effect of the HfO_2_ scale on oxygen diffusion at ultrahigh-temperature [44]. In addition, for the SI process, it is possible to increase the UHTCs content in the matrix using a vacuum vibration-assisted slurry impregnation process [45,46]. Hu et al. [47] combined vibration-assisted SI with low-temperature hot pressing and prepared a carbon fiber-reinforced ZrB_2_-SiC composite with uniformly distributed UHTCs, which exhibit strongly enhanced mechanical properties. Nevertheless, this method has not yet been used in the preparation of UHTCs-modified C/C composites.

In summary, while conventional UHTC modification methods (CVI, PIP, and RMI) each possess inherent drawbacks limiting their individual effectiveness, researchers overcome these limitations by employing synergistic combination processes. Furthermore, to enhance efficiency, pre-infiltration strategies—notably slurry infiltration (SI)—are increasingly adopted. SI allows controlled introduction of UHTC powders into porous substrates prior to densification, offering a cost-effective, flexible, and efficient pathway to optimize composite properties. The core of combined processes lies in “maximizing strengths but minimizing costs.” However, the optimization of process parameters in existing studies mostly relies on empirical trial-and-error, lacking theoretical guidance. We propose that the selection of combined processes should follow three principles: (1) minimization of fiber damage: When adopting high-temperature thermal processes such as RMI, a ceramic interlayer (e.g., ZiC) can be pre-introduced via PIP to isolate molten metals from carbon fibers (as validated in the study by Wen et al. [14]). (2) Balance between density and efficiency: For large-scale components, the combination of SI pre-filling and low-cycle CVI/PIP can shorten the densification cycle by over 40% while avoiding compositional segregation caused by single RMI. (3) Cost controllability: Compared with the pure CVI process, the raw material cost of the SI + PIP combination can be reduced by 30% (based on price comparisons of industrial-grade ceramic powders and precursors), making it more suitable for large-scale production. Future efforts should focus on establishing a quantitative relationship between process parameters, component dimensions, and performance indicators to realize “on-demand design” of combined processes.

## 3. Ablation Resistance and Mechanisms of UHTCs-Modified C/C Composites

### 3.1. Testing and Numerical Methods of the Ablation Resistance

**Figure 9:**
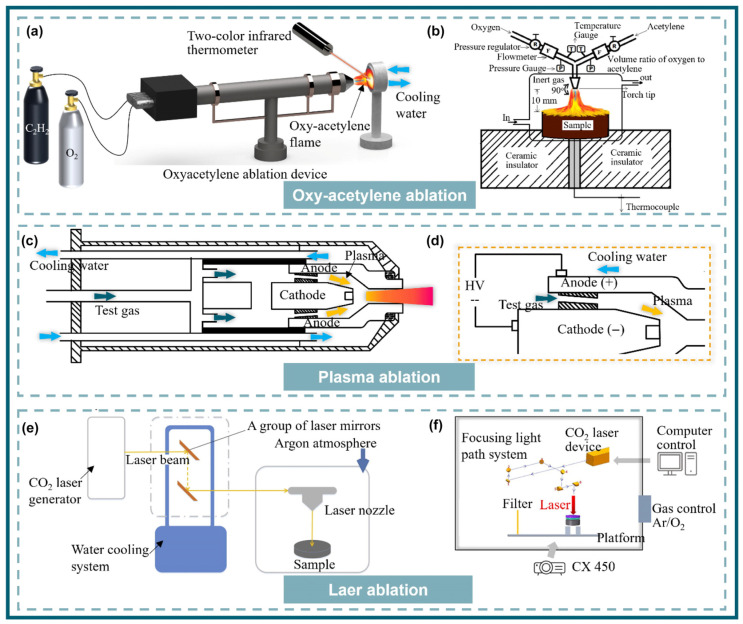
Schematic diagram of ablation testing systems and typical flame generation layout: (**a**,**b**) oxyacetylene ablation, (**c**,**d**) plasma ablation, and (**e**,**f**) laser ablation. Copied with permission from Elsevier [48].

To simulate the high-temperature oxidation and high-speed particle impact scenarios of UHTCs-modified C/C composites, researchers have proposed two testing methods. One method involves using high-temperature and high-speed flames or particle streams to impact the surface of the composites, assessing their static oxidation resistance, including oxyacetylene ablation, plasma ablation, and laser ablation (Figure 9) [48]. The other method aims to closely replicate the actual operating environment of the composites, which involves conducting wind tunnel tests under more complex conditions [49].

#### 3.1.1. Oxygen Acetylene Ablation

Oxygen acetylene was first used in welding, metal cutting, and other fields. It refers to the high temperature and high flame flow generated by acetylene combustion from the nozzle to the material surface. It has the advantages of simple equipment, convenient operation, and low cost. Oxygen acetylene ablation uses its high temperature and high flame flow to simulate the ablation environment. During the ablation process, the pressure caused by high temperature and high-speed airflow impacting the composite surface, the shear force generated when the airflow changes direction, and high temperature oxidation and heat conduction can better simulate the ablation environment of aircraft (Figure 9a). However, the oxygen acetylene ablation gas composition is relatively single, the concentration of flame flow is low, and the heat flow is not high, resulting in uneven pressure and temperature on the ablation surface [49].

#### 3.1.2. Plasma Ablation

Developed to overcome the issue of non-concentrated oxyacetylene flames, plasma ablation ionizes argon or nitrogen to produce plasma, accelerated through a nozzle to form a high-temperature (up to 3000 °C), high-speed flame stream (Figure 9b) [48]. The flame has highly concentrated heat and energy, though its speed is slower than oxygen–acetylene. Additionally, the high-temperature, high-speed N-containing gas may react with the metal surface of the composite materials to form nitrides, which can subsequently be oxidized [50,51].

#### 3.1.3. Laser Ablation

As lasers have evolved and found applications in the weapons industry, researching materials’ resistance to laser ablation has become a hot topic. This is also driven by the desire for higher energy and flow rate ablation equipment, making laser ablation a favored area of study among many researchers. Lasers can be categorized into CO_2_, fiber, and YAG types, each capable of delivering higher and more concentrated energy, reaching up to 100 MW·m^−2^ (oxygen–acetylene is approximately < 10 MW·m^−2^). Therefore, they can realistically simulate the extreme environments encountered by aircraft during operation [48,49,52,53,54].

#### 3.1.4. Wind Tunnel Ablation

Despite the different mechanisms of the three ablation methods, they all, to some extent, reflect the ablation resistance of the UHTCs-modified C/C composites. The drawbacks of these three ablation methods lie in the following: the composite material remains relatively stationary relative to the nozzle, ignoring the complex airflow around the vehicle during high-speed flight; furthermore, all three ablation methods heat up to the target temperature in an extremely short time, reflecting only the critical failure conditions of the composite material. However, the vehicle’s operational process involves a complex heating and temperature change process. To more realistically simulate the vehicle’s operating environment, wind tunnel experiments are widely used both domestically and internationally. A wind tunnel is a ducted test facility that uses artificially generated airflow to simulate total temperature, total pressure, Mach number, Reynolds number, and gas composition under the flight environment of the vehicle, serving as the ultimate means to evaluate the ablation resistance of hypersonic vehicles. Depending on the energy source, wind tunnels can be classified into air wind tunnels, methane gas wind tunnels, plasma wind tunnels, and arc-jet wind tunnels, and the most widely used for UHTCs-modified C/C composites are plasma wind tunnels [55,56,57] and arc-jet wind tunnels [58,59].

#### 3.1.5. Numerical Simulation

In addition to the four ablation methods mentioned above, numerical modeling and simulating the ablation/oxidation behavior of the UHTCs-modified C/C composites using different technologies/software (e.g., COMSOL, ANSYS Fluent) has attracted much attention in recent years because of its high efficiency, no damage to the samples, and relatively low cost [60,61,62,63,64]. Up to now, there are five methods to simulate the ablation and oxidation process of the UHTCs-modified C/C composites. (1) Molecular Dynamics (MDs) Simulations: (a) Ab Initio MD (AIMD): quantifies atomic-scale oxidation pathways and phase transitions. (b) Reactive Force Field MD (ReaxFF): simulates chemical bond breaking/formation during oxidation initiation. (2) Microstructure-Based Finite Element Modeling: (a) X-ray Computed Tomography-Driven Reconstruction (XCT): generates 3D finite element models from real microstructures to quantify thermal stress and ablation. (b) Representative Volume Element Models (RVEs): predict non-uniform ablation using stochastic algorithms and transient heat transfer analysis. (3) Multiphysics Coupling Frameworks: (a) Thermo-Chemo-Mechanical Models: couple heat transfer, chemical kinetics (e.g., Deal–Grove equation), and stress equilibrium to predict spallation. (b) Fluid–Ablation Interaction Models: integrate computational fluid dynamics (CFDs) with surface ablation responses for high-speed flow environments. (4) Fractal Geometry Approaches: (a) Weierstrass–Mandelbrot Interface Modeling: quantifies roughness-dependent oxygen diffusion using fractal dimension (Ds), height scaling (G), and frequency factor (γ). (5) Data-Driven Integrated Computational Materials Engineering (ICME): combines machine learning (e.g., neural networks), multiscale simulations, and experimental data to optimize composition/coating design. The key advantages of these modeling methods can be summarized as follows: (1) AIMD/ReaxFF: uncover atomic mechanisms. (2) XCT/RVE: capture real microstructure effects. (3) Multiphysics Models: resolve coupled ablation drivers. (4) Fractal Methods: parameterize complex interfaces. (5) ICME: accelerate material optimization. Nevertheless, because the structure of the UHTCs-modified C/C composites is rather complicated, the technology for the simulation of the ablation process should be further improved.

### 3.2. Evaluation Criteria of Ablation Resistance

Ablation is a complex physical and chemical reaction process. Under the impact of high-temperature, high-speed flame jets, the primary ablation mechanisms for UHTCs-modified C/C composites are chemical erosion and mechanical ablation. Chemical erosion mainly involves thermal physical reactions and high-temperature oxidation of the composite materials at high temperatures, while mechanical ablation results from the shear stress generated by the flame jet impacting the ablation surface, causing oxide shedding [65,66].

**Figure 10:**
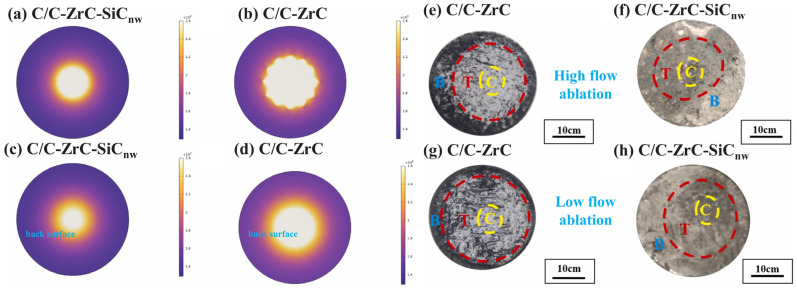
Temperature distribution predicted by thermal simulation in (**a**,**b**) ablated surface and (**c**,**d**) back surface, as well as macro appearance after ablation: (**e**,**f**) high flow ablation and (**g**,**h**) low flow ablation. Copied with permission from Elsevier [68].

After ablation, the surface is typically divided into three regions: central, transition, and edge. Ablation products and mechanisms differ across these regions [11,67]. The central region experiences direct impingement by the flame jet, with the highest thermal shock pressure and surface temperature, leading to the most severe oxidation ablation. For composites with poor ablation properties, oxides may flake off, forming deep ablation pits. The transition zone surrounds the central region, experiencing lower temperatures and thermal shock forces but significant shear stress from rebound expansion of the high-speed flame. Primary ablation products are various sintered oxides. The edge region is outermost, experiencing lower temperatures and pressures but potentially higher oxygen partial pressure, often forming a dense molten oxide film. Figure 10 shows the temperature simulation and surface morphology of the C/C composites modified with ZrC and SiC nanowires (SiC_nw_) during oxygen–acetylene ablation and after ablation, respectively [68]. As shown in Figure 10a,b, the temperature distribution of the ablation surface is clearly divided into three zones through simulation, with the sizes of these regions mainly related to the thermal conductivity of materials. The results show that the ablation temperature of the C/C-ZrC is higher than that of the C/C-ZrC-SiCnw under the same oxygen acetylene ablation, which is due to the high thermal conductivity of SiC_nw_. In Figure 10e–h, it can be seen that these composites form a relatively dense oxide layer after ablation, with distinct three regions. Further research indicates that the ablation products in the central region are ZrO_2_ layers, the transition region is a molten and dense SiO_2_ layer, and the edge region is a coexistence zone of SiO_2_ spherical particles and SiC nanowires.

The good or bad ablation performance is often evaluated by the linear ablation rate and mass ablation rate. During the ablation process, oxides will be produced on the surface, and some elements will volatilize, resulting in a change of thickness and mass of the sample before and after ablation. The calculation equations are shown as follows [68]:

(4) $$R_{l}=\frac{l_{0}-l_{1}}{t}$$

(5) $$R_{m}=\frac{m_{0}-m_{1}}{t}$$

Among them, *R_l_* represents the linear ablation rate, *R_m_* represents the mass ablation rate, *l*_0_ and *l*_1_, *m*_0_ and *m*_1_, respectively, represent the thickness and mass before and after ablation, and t represents the ablation time. In general, the smaller the linear ablation rate and mass ablation rate, the less the oxidation damage, and the better the material’s ablation resistance. Due to different preparation methods for C/C samples, the dimensions of the resulting composites vary; for example, the ablation surface size can be 29 mm or 20 mm, with different areas of oxidation damage, which leads to the mass ablation rate not being able to accurately evaluate the quality of ablation performance. Therefore, Equation (5) can be improved by introducing the surface area s of the ablation surface, thus better reflecting the actual ablation results, as shown in Equation (6) [69]:

(6) $$R_{m}=\frac{m_{0}-m_{1}}{t\cdot s}$$

In recent years, extensive research has been conducted on UHTCs-modified C/C composites. To enhance mechanical properties, oxidation resistance, and ablation performance while designing oxidation mechanisms, researchers have directed their attention to interfacial structure, preform density and pore distribution, and the content/distribution of UHTCs phases. The systems modified with UHTCs have evolved from single-phase ceramics like C/C-ZrC and C/C-HfC to duplex phases like C/C-ZrC-HfC and C/C-ZrB_2_-HfC, then to multiphase systems like C/C-ZrB_2_-HfC-SiC and C/C-ZrC-HfC-HfB_2_-SiC, and finally to the introduction of high-entropy ceramic phases. This progression has significantly reduced ablation rates and increased operating temperatures, promoting C/C composite application in aerospace. Table 2 lists recent studies on process, ablation conditions, and ablation performance of the UHTCs-modified C/C composites (detailed descriptions are provided in the following sections).

### 3.3. Single-Phase UHTCs-Modified C/C Composites

Research on UHTCs-modified C/C composites began with single-phase UHTCs, primarily metal carbides, borides, or nitrides of Zr, Hf, Ta, etc. These ceramics and their oxides have extremely high melting points. After ablation, they form protective layers adhering to the C/C surface, significantly enhancing ablation resistance. This section introduces single-phase UHTCs-modified composites based on Hf, Zr, Ta, and Ti systems.

#### 3.3.1. Zr-Based UHTCs-Modified C/C Composites

**Figure 11:**
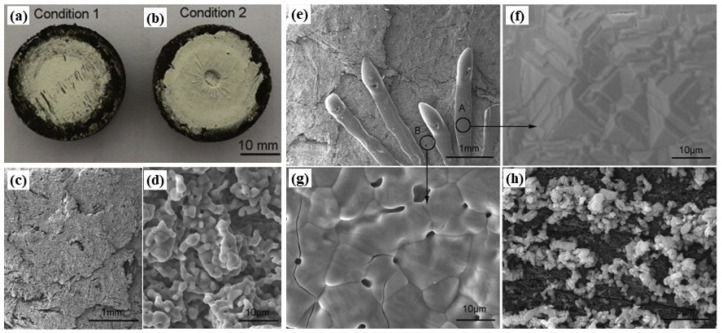
Photographs and SEM images of the C/C-ZrC composites after ablation under different conditions: (**a**,**b**) surface appearance of the ablated C/C-ZrC composites; surface morphology of C/C-ZrC composites after ablation under condition 1: (**c**) low magnification and (**d**) high magnification; surface morphology of C/C-ZrC composites after ablation under condition 2: (**e**) low magnification (**f**,**g**) high magnification and (**h**) undersurface oxidation composites. Copied with permission from Elsevier [70].

ZrC is the most classic and commonly used single-phase UHTC, with low density (ZrC: 6.6 g/cm^3^, TaC: 14.5 g/cm^3^, HfC: 12.7 g/cm^3^), thermal expansion coefficient, and cost, and it has outstanding ablation resistance, making it the first UHTC applied to modified C/C composites [25,41,42,51,84]. During ablation at temperatures higher than ca. 1800 °C, ZrC can form relatively dense ZrO_2_, but when the ablation temperature is lower, the high-melting-point ZrO_2_ (melting point: 2700 °C) forms a framework that cannot promptly compensate for defects (e.g., cracks and pores) caused by oxidation. The onset temperature for ZrC oxidation is around 800 °C [85]. The oxidation of ZrC leads to significant volume change and CO/CO_2_ release, which cause lots of cracks and pores in the ZrO_2_ scale, respectively. When the oxidation temperatures are higher than ca. 800 °C but lower than ca. 1800 °C, the generated ZrO_2_ cannot be densified due to its high softening temperature. Therefore, no self-sealing effect can be obtained in this temperature range. In addition, the thermal expansion coefficient mismatch between the ZrC matrix and ZrO_2_ scale also results in cracks at high temperatures. Under this condition, these defects are the oxygen diffusion channels, leading to a decrease in protective capability. Liu et al. [69] used ICVI combined with RMI to prepare C/C-ZrC composites. After 60 s of oxyacetylene ablation, the mass ablation rate was 0.422 mg/s/cm^2^, attributed to the formation of relatively dense ZrO_2_, which reduced the oxidation of PyC and carbon fibers. Shen et al. [65] prepared C/C-ZrC composites with different ZrC contents using SI and ICVI (no coating was applied in this case). After 2500 K oxyacetylene ablation, they found that the linear ablation rate and mass ablation rate of C/C composites with 4.14 wt.% ZrC content was 5.1 × 10^−4^ mm/s and 6.2 × 10^−4^ g/s, respectively, representing an 87.0% and 77.0% decrease compared to unmodified C/C composites. Li et al. [70] prepared C/C-ZrC composites via the PIP process. The ablation performance of the composites was investigated by oxyacetylene flame with heat flux of 2.38 MW/m^2^ (Figure 11a, condition 1) and 4.18 MW/m^2^ (Figure 11b, condition 2), respectively. The results showed that C/C-ZrC composites exhibited excellent ablation resistance under condition 1 for 120 s (Figure 11a), and the linear ablation rate and mass ablation rate of the material were −1.23 × 10^−3^ mm/s and −1.64 × 10^−3^ g/s, respectively. A tree coral-like ZrO_2_ protective layer formed after ablation (Figure 11c,d). However, when the heat flux increased to 4.18 MW/m^2^ (condition 2), the maximum temperature of the ablated surface reached 2500 °C (even 3000 °C in the center), and a strong degradation of ablation resistance was observed (Figure 11b). The main reason for the degradation of ablation resistance was attributed to the spalling of the formed ZrO_2_ layer as the temperature reached as high as 2500 °C, and it could not provide effective protection to the substrate. Particularly, at the center of the samples, the surface temperature increases to 3000 °C. The ZrO_2_ (melting point 2680 °C) began to melt and was dispersed under high-speed flame impact (Figure 11e–g). After peeling off the surface ZrO_2_ layer, it can be found that the same ZrO_2_ grains adhered to the carbon matrix (Figure 11h), which protects the composites from ablation to a certain extent.

The excellent protective effect of ZrB_2_ stems not only from the formation of a glassy ZrO_2_ protective layer but also from the rapid volatilization of low-melting-point B_2_O_3_ (ca. 445 °C). This volatilization absorbs significant heat (“endothermic cooling effect”), effectively reducing the surface temperature and inhibiting carbon phase oxidation. Additionally, the volatilization of B_2_O_3_ minimizes the residual liquid oxide, preventing the failure of the protective layer due to the loss of low-melting-point phases (such as pure B_2_O_3_). Tong et al. [86] prepared C_f_/ZrB_2_ preforms using a weaving-powder-laying method and fabricated C/C-ZrB_2_ composites through an “impregnation-carbonization” process. After plasma ablation for 120 s, the mass ablation rate and linear ablation rate were 0.30 mg/s and 8.75 μm/s, respectively, significantly improved compared to C/C composites (mass ablation rate 2.23 mg/s and linear ablation rate 15.25 μm/s). This improvement is attributed to the oxygen-blocking effect of the glassy ZrO_2_ and the synergistic effect of B_2_O_3_ volatilization and heat absorption. After 10 min and 60 min of oxidation at 1100 °C, the mass ablation rates were 2.67% and 20.47%, respectively. The flexural strength remained at 80% of its pre-oxidation value (from 81.1 MPa to 64.4 MPa) after 10 min of oxidation and both before and after oxidation exhibited pseudoplastic fracture modes. Sun et al. [87] used ultrasonic and vacuum immersion–carbon thermal reduction to introduce ZrB_2_ into carbon preforms, preparing ZrB_2_-doped C/C composites via TG-CVI and graphitization densification processes. After oxygen–acetylene ablation tests, composites with 6.87 wt% ZrB_2_ showed *R_l_* reduced by 64.9% and *R_m_* by 67.5%. Ablation is primarily controlled by thermo-chemical and thermo-physical reactions, with minimal mechanical ablation. During the ablation process, the evaporation of ZrO_2_/B_2_O_2_ removes a significant amount of heat, reducing the thermal impact of the flame on the ablation surface. Yang et al. [88] conducted oxygen–acetylene ablation tests on C/C-ZrB_2_ composites with different ZrB_2_ volume fractions, confirming the positive role of ZrB_2_ in enhancing the ablation resistance of composites. The parameters were set at a flame temperature of 3000 °C and an ablation time of 60 s. The experimental results showed that the best-performing 10 vol% sample achieved R_m_ = 0.01802 g/s and R_l_ = 0.0122 mm/s. In addition, the text points out that ZrB_2_ undergoes an oxidation reaction at high temperatures to form ZrO_2_ and B_2_O_3_. Among these, B_2_O_3_ rapidly evaporates above 1200 °C, while ZrO_2_ has a melting point exceeding 2700 °C. The solid or highly viscous liquid protective layer formed on the material’s surface effectively hinders oxygen diffusion and reduces the impact of high-speed airflow. However, excessive ZrB_2_ content weakens interlayer bonding and increases porosity due to significant volatilization of B_2_O_3_, causing surface shedding under airflow and increasing ablation rate. Actually, the ZrB_2_ is always used together with SiC for ultrahigh-temperature applications because the B_2_O_3_ can react with SiO_2_ to generate less volatile borosilicate glass with low permeability for oxygen [89,90].

#### 3.3.2. Hf-Based UHTCs-Modified C/C Composites

HfC, with its extremely high melting point (~3959 °C), very low vapor pressure, excellent ablation resistance, and chemical inertness, is often used as a modifier for C/C composites in [25]. Its oxide HfO_2_ has an exceptionally high melting point (~2900 °C) and a relatively low vapor pressure (~3 × 10^−5^ atm). HfC is a strong candidate for anti-ablation materials in aerospace applications. The reason it enhances ablation resistance lies in the layers that form after ablation: From the substrate to the outer layer, there are the dissolved oxygen HfC layer, the dense carbon oxide HfC_x_O_y_ layer, and the porous HfO_2_ layer. Among these, HfC_x_O_y_ serves as an excellent thermal barrier and oxygen diffusion barrier [25,49], preventing further oxygen and oxygen atoms from diffusing inward.

**Figure 12:**
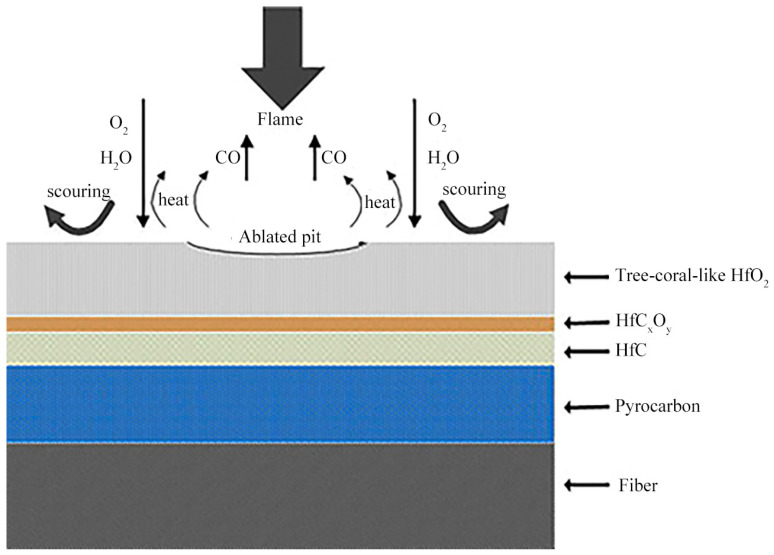
Schematic diagram of the layered structure of C/C-HfC composites after ablation. Copied with permission from Elsevier [73].

In the early stages of research, researchers considered using RMI to introduce Hf, but due to its high melting point and the tendency of Hf infiltration to easily damage carbon fibers, it was not given much attention. With the production and use of ultrahigh-temperature-strength carbon fibers and breakthroughs in the research of Hf-containing precursors and Hf-containing ceramic powders, the use of Hf-modified C/C composites has attracted widespread interest among researchers [75,91,92]. Xue et al. [73] successfully prepared C/C-HfC composites with a density of 2.01 g/cm^3^ via PIP, which underwent plasma ablation for 240 s with a surface temperature exceeding 2573 K. The linear (R_l_) and mass (R_m_) ablation rates were 5.31 μm/s and 0.55 mg/cm^2^/s, respectively, representing reductions of 71% and 86% compared to C/C composites. Figure 12 shows a schematic diagram of the surface oxidation layer after ablation of C/C-HfC composites, indicating that the improvement in ablation resistance is attributed to the formation of tree coral-like HfO_2_ oxide layers and the thermal blocking effect of HfC oxidation and ablation gas products on oxygen consumption. Additionally, the dense HfC_x_O_y_ phase serves as a thermal barrier and oxygen diffusion barrier, thus significantly enhancing the anti-ablation performance of C/C composites.

However, excessive HfC may not be beneficial. This is related to the coefficient of thermal expansion (CTE) mismatch between HfC and carbon fibers, carbon matrix, as well as the PyC interface. The CTE of HfC is around 6.6 × 10^−6^ K^−1^ [93], which is in sharp contrast to carbon fibers (−0.5–1.0 × 10^−6^ K^−1^) [94], polymer-derived carbon matrix (2–4 × 10^−6^ K^−1^) [95], and pyrolytic carbon (PyC) interface (4–6 × 10^−6^ K^−1^) [96]. If the HfC content is too low, the super-high-temperature ceramic modification effect is minimal. If the HfC content is too high, not only will cracks form due to the significant difference in CTE with the C matrix and fibers, but it will also excessively consume or damage the PyC interface and even the carbon fibers. This is because, in the PIP process, the PyC interface and the carbon fibers may be consumed by the oxygen-containing HfC precursors and their oxide derivatives [73]. Compared with the PIP process, the consumption of carbon phases in the RMI process is more serious. This is because the HfC/ZrC are generated by reactions between Hf/Zr-containing melt (e.g, Hf, Zr, Hf-Si, and Zr-Si) and carbon phases (incl. carbon matrix, PyC interface, and carbon fibers) in the C/C composites [14,97]. Pyrolytic carbon layer and even the carbon fibers Feng et al. [98] studied the impact of the PyC interface on the mechanical and ablation properties of C/C-HfC composites, finding that the PyC interface can effectively protect fibers from damage during the carbon thermal reduction reaction in the PIP process and mitigate the oxidation of carbon fibers during ablation. Li et al. [74] prepared C/C composites with different HfC contents and found that when the HfC content is around 6.5 wt.%, they exhibit excellent ablation resistance. This is because, as the HfC content increases, the phonon-defect interactions generated by CO thermal motion in HfO_2_ micropores and interstices enhance the thermal conductivity of the materials. The generated HfO_2_ and HfC_x_O_y_ act as oxygen barriers, thus improving ablation resistance. When the HfC content exceeds 6.5 wt.%, cracks caused by thermal expansion differences become diffusion channels for the oxidizing atmosphere, accelerating the ablation of the composite material.

**Figure 13:**
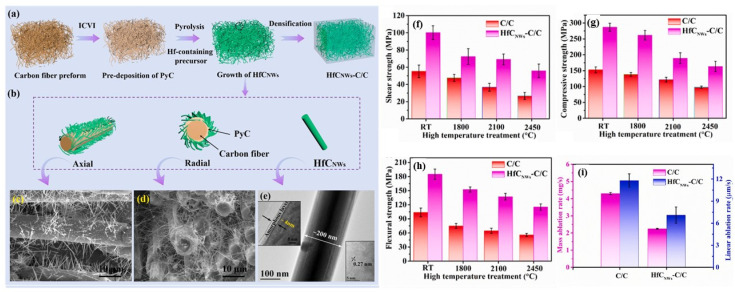
(**a**,**b**) Schematic diagram of the process of HfC_NWs_-C/C; (**c**,**d**) SEM images of the as-prepared HfC_NWs_ in carbon fiber preforms; (**e**) TEM images of the HfC_NWs_; (**f**–**h**) mechanical properties of the composites before and after heating at 1800 °C, 2100 °C, and 2450 °C, respectively: (**f**) interlaminar shearing strength (ISS); (**g**) out of plane compression strength (OCS); and (**h**) the flexural strength (FS); and (**i**) ablation performance of the C/C and HfC_NWs_-C/C composites. Copied with permission from Elsevier [99].

Another direction to improve ablation resistance is to enhance its thermo-physical properties, such as thermal conductivity, thermal expansion coefficient, and mechanical bonding performance. Research has found that preparing HfC nanowires (HfC_nw_) is an effective method [75,96,99]. Tian et al. [96] prepared HfC nanowire-modified C/C-HfC_nw_ composites through catalytic CVD and hot pressing, increasing the thermal conductivity in the *Z*-axis direction from 100 °C to 2500 °C and reducing CTE by 24%. After 20 s of oxygen–acetylene ablation, the linear ablation rate and mass ablation rate were 21.0 μm/s and 5.3 mg/s, respectively. Further analysis revealed that compared with unmodified C/C composites, the oxidation of HfC nanowires and the melting and evaporation of their oxides can consume a large amount of heat, thereby lowering the ablation surface temperature. On the other hand, the lower CTE of HfC_nw_-C/C composites helps improve dimensional stability during ablation. Additionally, the HfO_2_ fiber network formed by the oxidation of HfC_nw_ acts as a protective layer to some extent, alleviating oxidation, thermal erosion, and mechanical erosion. Fu et al. [99] prepared a hafnium carbide nanowire (HfC_NWs_) modified C/C composite using a combined Ni-assisted PIP and ICVI process (Figure 13a–e). Their high-temperature performance in terms of mechanical properties and ablation resistance is systematically investigated as well. As shown in Figure 13f–h, with the presence of HfC_NWs_, the interlaminar shearing strength (ILSS), out-of-plane compression strength (OCS), and flexural strength (FS) of the HfC_NWs_-C/C composites increased by 81.36%, 87.96% and 78.38%, respectively. The mechanical strength retention rate of the HfC_NWs_-C/C composites is also superior to that of pure C/C composites. The corresponding mass and linear ablation rates are illustrated in Figure 13i. The mass and linear ablation rates of the HfC_NWs_-C/C composites are obviously lower than those of the C/C composites, which suggests that the HfC_NWs_-C/C composites provide a potential possibility to be applied in extreme conditions.

Regarding HfB_2_, its oxidation mechanism is similar to that of ZrB_2_, primarily due to the excellent pore-filling and volatile heat absorption effects of B_2_O_3_ formed through oxidation. Zhang et al. [76] prepared C/C-HfB_2_ composites using the PIP combined with CVI. At an oxygen–acetylene ablation heat flux of 2.38 MW/m^2^, the C/C-HfB_2_ composite showed good ablation performance, with a HfO_2_ layer on the surface effectively blocking heat transfer and the erosion by the oxygen–acetylene flame. The linear ablation rate after 90 s was 3.14 × 10^−3^ mm/s, and the mass ablation rate was 0.121 mg cm^−2^ s^−1^. When the heat flux increased to 4.18 MW/m^2^, the HfO_2_ layer cracked and peeled off, leading to a decrease in ablation performance. Thermal retardant ability tests showed that at heat fluxes of 2.38 MW/m^2^ and 4.18 MW/m^2^, the thermal resistance of the C/C-HfB_2_ composites was 308 °C/mm and 332 °C/mm, respectively. In addition, Li et al. investigate the effects of HfB_2_ content on the ablation rates of the C/C-HfB_2_ composites [100]. They found that, compared with the oxidation behavior of carbon fibers within the C/C composites, the beginning oxidation temperature of HfB_2_-coated carbon fibers in the C/C-HfB_2_ composites is raised from 300 to 700 °C. The composites with HfB_2_ content below 10 wt.% exhibit better ablation resistance than those with HfB_2_ content between 10 and 15 wt.%, while the composites with HfB_2_ content above 15 wt.% exhibit more outstanding ablation resistance. For the composites with 18.59 wt.% HfB_2_, the linear and mass ablation rates are 6.83 × 10^−3^ mm/s and 2.77 × 10^−3^ g/s, which decreased by about 51.80% and 24.32%, respectively, compared with C/C composites without HfB_2_. This is because the HfB_2_ phase is able to strengthen the interface between carbon fibers and matrix and protect carbon fibers against oxidation and ablation.

#### 3.3.3. Ta-Based UHTCs-Modified C/C Composites

TaC, a typical UHTC with an extremely high melting point (3983 °C), is suitable for extremely high-temperature environments. Its C/C matrix compatibility with HfC (6.3 × 10^−6^ K^−1^) is slightly improved to 6.5 × 10^−6^ K^−1^. The ablation rate of carbon fibers by the oxide phases produced during ablation (such as liquid Ta_2_O_5_) is significantly reduced, less than 0.01 mm/s, while for HfO_2_, it is 0.03 mm/s. A key feature of TaC is its self-healing capability for micro-cracks in the oxide layer. Chen et al. [101] conducted a detailed study on the high-temperature oxidation process of CVI-TaC-C/C materials. The protective barrier formed during the high-temperature oxidation of TaC consists of Ta_2_O_5_, followed by dense TaC_x_O_y_ layers and the matrix TaC. In the early stages of formation, Ta_2_O_5_ cannot block oxygen and has numerous micro-cracks in its structure. During oxidation, matrix expansion merges cracks, while the glassy Ta_2_O_5_ further seals them. Experimental results show that after about 10 min of initial linear expansion, TaC can achieve self-healing of cracks except for those caused by thermal stress. Apart from the mechanical barrier provided by the dense Ta_2_O_5_ layer, there is a transition layer of about 1 μm of TaC_x_O_y_ between the ceramic and the matrix. Oxygen is trapped at lattice points and cannot diffuse freely, which further prevents O_2_ from corroding inward along the oxide layer. After crack repair, the oxidation rate is limited by O diffusion in the transition layer. Djugum et al. [102] performed a 2 MW/m^2^ oxyacetylene flame linear ablation test on dense TaC-C/C honeycomb woven components. During the experiment, the peak center temperature was approximately 1750 °C. Protected by glassy Ta_2_O_5_, the ablation rate (0.0057 mm/s) was much lower than pure C/C (0.0132 mm/s). Yang et al. [88] conducted oxyacetylene ablation tests on C/C-TaC composites with different TaC volume fractions, confirming that TaC has excellent ablation protection. The parameters were set to a flame temperature of 3000 °C and an ablation time of 60 s. The experimental results showed that the best-performing 15 vol% sample achieved a R_m_ of 0.02510 g/s and a R_l_ of 0.0290 mm/s. Notably, the paper also pointed out that Ta_2_O_5_ has a lower melting point (1872 °C), and pure TaC ceramics are more susceptible to erosion in complex gas environments.

#### 3.3.4. Ti-Based UHTCs-Modified C/C Composites

TiC is a commonly used UHTC in lightweight design, with a density of 4.9 g/cm^3^, significantly lower than ZrC (6.6 g/cm^3^) and HfC (12.7 g/cm^3^). Its melting point is 3100 °C, and its vapor pressure is relatively low (~10^−7^ atm at 1800 °C), allowing it to maintain structural stability at high temperatures [103]. During ablation, the TiO_2_ ceramic phase formed from TiC oxidation has a high viscosity and melting point (1843 °C), forming a continuous and dense protective film. It exhibits some fluidity in the mid-temperature range (1000–1600 °C) and can dynamically repair ablation defects. In the temperature range of 2000–2400 °C, it shows excellent erosion resistance. TiC is often used in conjunction with SiC or with other UHTCs by forming solid solutions [23,81,104,105], while C/C-TiC composites are less common. Although there are some related patents, most focus on its mechanical properties rather than its ablation resistance [103].

### 3.4. Multiphase UHTCs-Modified C/C Composites

Studies revealed that single-phase UHTCs modification offers limited ablation resistance improvement over unmodified C/C composites. At lower temperatures (<1000 °C), the UHTCs phase is prone to oxidation and cracking, offering limited protection. At higher temperatures, there is a significant difference in thermal expansion coefficients between UHTCs and their oxides with the C/C matrix, making it easy for porous oxide layers to form and crack off during ablation, resulting in even poorer ablation resistance than unmodified C/C composites [70,76,100,106,107,108]. In fact, the operating temperatures of hypersonic vehicles and re-entry atmospheric flight vehicles vary continuously between 1000 °C and 3000 °C, requiring thermal protection materials with short processing times, low density, high-temperature resistance, high strength and toughness, good long-term oxidation resistance and ablation durability, and a long service life [109,110]. To overcome single-phase limitations and further enhance performance, researchers try to introduce the second or third phase (e.g., SiC, MoSi_2_, and [111]) to form multiphase UHTCs-modified C/C composites, leveraging their differing oxidation resistance properties for broader thermal protection [112,113,114,115,116].

The most typical example is the combination of UHTCs and SiC to modify C/C composite materials. With years of development history, C/C-SiC composites exhibit excellent high-temperature oxidation resistance, low thermal expansion coefficient, and superior friction and wear properties. As the new generation of high-performance braking materials succeeding powder metallurgy and C/C composites, they are widely applied in aerospace components such as engine exhaust nozzles, thrust chambers, combustor–nozzle assemblies, and braking systems [117,118,119,120]. During medium–low temperature ablation, SiC forms a dense SiO_2_ film in the glass phase, which significantly compensates for surface defects of the matrix and provides excellent protection even at 1600 °C, making it an ideal medium to low-temperature thermal protection material. Adding SiC to UHTCs-modified C/C offers several advantages: (1) Si can form alloy compounds with Zr and Hf, making it possible to prepare C/C-SiC-UHTCs through rapid and low-cost RMI. For example, the melting point of metallic Zr is 1855 °C, while Zr-Si can form two eutectic alloys with lower melting points—Zr_9_Si_1_ (melting point: 1570 °C) and Zr_1_Si_9_ (melting point: 1370 °C)—allowing for RMI processes at lower temperatures; (2) the thermal expansion coefficients of SiC are close to those of C/C, significantly lower than those of UHTCs and their oxides, preventing micro-cracks or damage to carbon fibers during composite preparation; (3) during ablation, SiO_2_ exhibits excellent fluidity, which helps compensate for defects caused by oxidation of UHTCs, addressing issues of oxide cracking and delamination from the matrix; and (4) the formation of SiO_2_-UHTCs oxide symbiotic structures or silicates further resists the erosion of flame jets and oxygen penetration, thus better protecting the C/C matrix [71,121].

In recent years, researchers have conducted extensive studies on C/C-SiC-UHTCs, analyzing the effects of C/C density, pore distribution, SiC and UHTCs content, particle size, and distribution on ablation performance, achieving good results. Li et al. [112] selected a C/C matrix with different initial densities and prepared C/C-ZrC-SiC composites using the PIP process. They found that the low-density C/C matrix had a lower final density and higher porosity after PIP, while the high-density C/C matrix introduced less ZrC and SiC ceramics during the PIP process. Therefore, only a moderately dense C/C matrix with appropriate pore distribution and ceramic content exhibits better ablation performance. He et al. [122] also found C/C matrix with appropriate density (e.g., 0.91 g/cm^3^) yielded C/C-ZrC-SiC with dense microstructure and suitable ceramic content, showing excellent ablation resistance. Tang et al. [44] used slurry impregnation to introduce HfB_2_, preparing C/C-HfB_2_-SiC composites. After ablation at 2500 °C for 120 s, the mass ablation rate was only 0.5 mg/s, thanks to the complementary effects of the two oxides. Zhao et al. [71] prepared C/C-ZrC-SiC composites via RMI, showing improved R_l_ and R_m_ compared to C/C-ZrC. The ablation morphology reveals the formation of SiO_2_-ZrO_2_ symbiotic structures, where SiO_2_ fills the defects in ZrO_2_, thus enhancing ablation performance. In fact, according to the SiO_2_-ZrO_2_ binary phase diagram, there is a eutectic point between these two phases, leading to the formation of ZrO_2_-SiO_2_ binary symbiotic structures [121]. This means that solid-phase ZrO_2_ pinches off molten SiO_2_, preventing it from being washed away by high-speed flame jets, while SiO_2_ can fill the defects in ZrO_2_ particles or clusters. Therefore, this symbiotic structure improves the ablation resistance of the C/C-ZrC-SiC composites.

**Figure 14:**
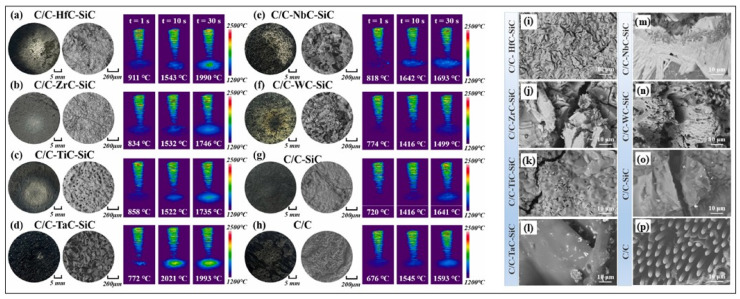
(**a**–**h**) Macroscopic and infrared imaging of the C/C-MeC-SiC composites during ablation as well as (**i**–**p**) SEM images of the surface morphology of the C/C-MeC-SiC composites after ablation for 30 s under the plasma torch with the heat flux density of 7.26 MW/m^2^, with (**a**,**i**) C/C-HfC-SiC, (**b**,**j**) C/C-ZrC-SiC, (**c**,**k**) C/C-TiC-SiC, (**d**,**l**) C/C-TaC-SiC, (**e**,**m**) C/C-NbC-SiC, (**f**,**n**) C/C-WC-SiC, (**g**,**o**) C/C-SiC, and (**h**,**p**) C/C. Copied with permission from Elsevier [60].

Zhang et al. fabricated a series of C/C-MeC-SiC composites (where Me represents Hf, Zr, Ti, Ta, Nb, and W) using the RMI method and investigated their ablation-resistant behaviors and mechanisms by both theoretical calculations and experimental verification [60]. It was observed that for composites containing HfC and ZrC (i.e., C/C-HfC-SiC and C/C-ZrC-SiC), a robust ablation-resistant framework with high melting points was formed after ablation, consisting of a skeletal structure. However, the absence of low-melting-point phases led to a loose, porous morphology (see Figure 14a,b,i,j). The high fluidity of the oxidation products of the TiC system (C/C-TiC-SiC) can rapidly densify the oxide layer, while the volatilization of gaseous by-products leads to the honeycomb-like structure in the oxide layer (Figure 14c,k). WC exhibits excellent resistance to high-temperature gas flow erosion (0.09 mg/s). However, the volatility of WO_3_ limited its application in long-term ablation. Needle-like Nb_2_O_5_ phases formed on the surface of the ablated C/C-NbC-SiC composites (Figure 14m), which have the potential to quickly fill porous structures under a thermal shock environment. Based on this research, some strategies have been proposed for the modification of C/C composites with UHTCs. Firstly, the sintering capability of the ceramics formed upon reaction with the carbon matrix requires attention. This is because achieving a densified ceramic layer is essential for effective scouring resistance. To promote this densification, incorporating suitable sintering additives into the reaction process proves beneficial. Secondly, maintaining the stability of phase structures under high-temperature conditions is equally critical. Generally, this relies on the interaction between high-melting-point skeletal supports and high-fluidity oxides, while minimizing excessive gas by-products. The last but not least point is thermal matching between the ceramic and the matrix, which helps to avoid cracking caused by excessive thermal stress [60].

Given the success of SiC-containing biphasic UHTCs-modified C/C composites, researchers began exploring triphasic and tetra-phasic composites. Unlike biphasic systems, multiphase UHTCs form more complex oxide layers or borosilicate glass layers, thus offering better thermal protection performance. Huang et al. [77] prepared C/C-ZrB_2_-ZrC-SiC using C_f_/ZrB_2_ preforms. After plasma ablation, improved resistance was attributed to the SiO_2_-ZrO_2_ barrier layer, high-temperature Zr-Si-O glass layer, and heat dissipation from gas product evaporation. Yang et al. [123] prepared C/C-ZrB_2_-ZrC-SiC composites via full PIP and studied their mechanisms under oxygen–acetylene and plasma ablation. The results showed that under oxygen–acetylene conditions, a more intense process formed a molten SiO_2_-ZrO_2_ film, while under plasma ablation, a porous layer composed of molten ZrO_2_-SiO_2_, ZrB_2_-ZrC, and SiC particles formed, demonstrating good ablation performance. Meng et al. [124] prepared C/C-HfC-HfB_2_-SiC composites, which had a mass ablation rate of only 1.22 × 10^−6^ g/cm^2^/s and a linear ablation rate of 1.33 × 10^−5^ mm/s, when ablated for 600 s at 2300 K in an arc wind tunnel (with 5% water content). Chen et al. [78] prepared C/HfC-ZrC-SiC composites with different HfC contents by RMI. At 8.8 wt.% HfC, a dense HfO_2_-ZrO_2_-SiO_2_ oxide layer formed, preventing SiO_2_ evaporation. And the mass ablation rate was only 1.5 mg/s. Makurunje et al. [125] prepared C/C-SiC-TiC-TaC (C/C-STT) composites using the melt infiltration method and systematically studied their oxidation behavior in air at 1873 K and ablation mechanisms under oxygen–acetylene flames (4 MW/m^2^). After 480 s at 1873 K in air, the mass lablation rate of C/C-STT was one order of magnitude lower than that of C/C, and it entered a mass gain regime after 240 s. This is attributed to the formation of a glass–ceramic coating (containing SiO_2_, TiO_2_, and Ta_2_O_5_ eutectics) that fills pores and forms a dense oxide layer. After 7.5 s oxyacetylene ablation, the mass ablation rate (3.9 mg/s) and linear ablation rate (2.2 μm/s) of the C/C-STT were much lower than those of C/C composites. A four-layer gradient structure formed on the surface, with the third layer achieving crack self-healing through liquid SiO_2_-Ta_2_O_5_ eutectics, significantly delaying the ablation process. The core mechanism of the ablation process is as follows: SiC oxidation forms the SiO_2_ glass phase, (Ta, Ti) C oxidation forms (Ti, Ta)O_2_ and Ta_2_O_5_, which work together to form a highly viscous glass-ceramic. Zhang et al. [63], based on the ablation mechanisms of the C/C-MeC-SiC (Me: Hf, Zr, Ti, Ta, Nb, and W) composites, prepared C/C-HfC-ZrC-TaC-SiC composites, which exhibit improved ablation resistance with mass and linear variation rates of 0.175 mg/s and 0.701 μm/s, respectively, after long-time ablation (1000 s, 40 s × 25 cycle) under oxyacetylene torch (4.2 MW/m^2^). Shen et al. prepared rare earth-modified C/C-ZrC-SiC composites using molten salt-assisted RMI. Studies have shown that rare earth fluorides YF_3_ can fill internal micro-pores and cracks, increasing the overall density of the material and reducing porosity. During ablation, the oxidation-formed Y_2_O_3_ can stabilize ZrO_2_, reducing CTE differences, enhancing matrix bonding, and forming a dense oxide layer that combines well with the underlying matrix material, effectively improving oxidation and ablation resistance. The mass ablation rate and linear ablation rate of YF_3_ modified C/C-ZrC-SiC composites are reduced by 42.61% and 90.14%, respectively [126,127].

**Figure 15:**
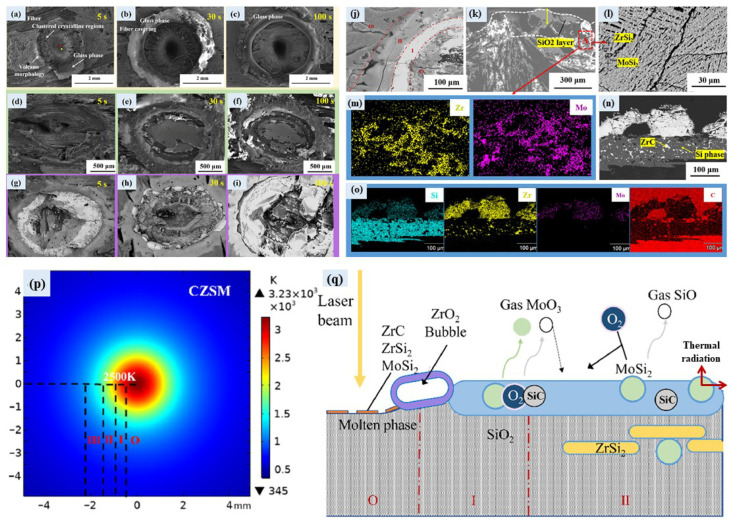
Ablation morphologies, microstructure, temperature distribution, and ablation mechanisms of the ablated composites: (**a**–**c**) 5, 30, and 100 s for CS; (**d**–**f**) 5, 30, and 100 s for CZS; (**g**–**i**) 5, 30, and 100 s for CZSM; (**j**) surface and regions of the ablated CZSM, (**k**) cross-sectional view of area A, (**l**) large view of area A, (**m**) element mapping of area A, (**n**) molten pool morphology, and (**o**) element mapping of the molten pool; (**p**) temperature distribution of CZSM during ablation for 100 s; and (**q**) ablation mechanisms of the CZSM composites. Copied with permission from Elsevier [113].

In addition, Geng et al. [113] prepared C/C-SiC (denoted as CS), C/C-SiC-ZrC (denoted as CZS), and C/C-SiC-ZrC-MoSi_2_ (CZSM) composites using the RMI process and conducted ablation tests in an atmospheric environment with a CO_2_ laser (500 W, 2 mm spot). Ablation morphologies and temperature distribution of the composites are shown in Figure 15a–o and Figure 15p, respectively. After ablating for 100 s, the calculated surface temperature of the CZSM is ca. 3000 °C (Figure 15p), which is relatively lower than that of CS (ca. 4035 °C) and CZS (ca. 3955 °C). The ablation depth of the C/C-SiC-ZrC-MoSi_2_ composite is only 1 mm after ablation for 100 s, while the ablation depth of the C/C composites is larger than 2.5 mm. The main principles behind the improved ablation performance of this material are as follows: (1) MoSi_2_ promotes the formation of a dense SiO_2_ glass layer, inhibiting oxygen diffusion; (2) at low temperatures (<1100 °C), the introduction of MoSi_2_ reduces the thermal diffusivity and thermal conductivity of the materials, decreasing heat transfer to the interior; and (3) MoSi_2_ has a high emissivity, dissipating heat through radiation at high temperatures and slowing down the ablation process. Moreover, MoSi_2_ also influences the oxidation behavior of the ZrC-SiC system, maintaining a low oxygen partial pressure environment for the oxidation of ZrC. The ablation mechanisms of the C/C-SiC-ZrC-MoSi_2_ composites are shown in Figure 15q.

Based on the studies mention above, the role of SiC in multiphase systems is not merely “defect filling” but the construction of a “dynamic protection system”: At medium–low temperatures (1000–1600 °C), the SiO_2_ glass phase flows rapidly to seal cracks in ZrO_2_/HfO_2_; at higher temperatures (>2000 °C), SiO_2_ forms a low-melting eutectic phase with ZrO_2_/HfO_2_ (e.g., melting points of ZrSiO_4_ is 1687 ± 10 °C [128]), which, although reducing skeleton strength, consumes flame kinetic energy through “sacrificial flow.” This dual role dictates that SiC content should be controlled within 10–30 vol%. Insufficient content fails to form a continuous glassy phase, while excessive content leads to excessive mass loss at ultrahigh-temperatures. Furthermore, we note that the synergistic effects between SiC and different UHTCs vary. Specifically, the difference in CTE between SiC and HfC/ZrC is smaller than that between SiC and TiC, which is more conducive to preventing crack initiation (β-SiC: 3.8 × 10^−6^ K^−1^, HfC: 6.6 × 10^−6^ K^−1^, ZrC: 6.7 × 10^−6^ K^−1^, and TiC: 7.4 × 10^−6^ K^−1^) [93]. This necessitates consideration of multiple factors, such as compatibility during the compositional design of multiphase systems.

### 3.5. Multicomponent UHTCs-Modified C/C Composites

While the ablation performance of multiphase UHTCs-modified C/C composites has been improved, researchers have also identified significant issues. Specifically, there is a sequence in the introduced processes for multiphase UHTCs. During high-temperature ablation, different oxides form due to varying melting points. For example, low-melting-point phases like SiC oxidize first, followed by high-melting-point materials such as ZrC and HfC, forming layered structures. Moreover, the thermal expansion coefficients of the oxide products differ significantly, and molten SiO_2_ may not be able to promptly fill defects, leading to cracking of the oxide layers. This unstable oxidation phenomenon and complex oxidation mechanisms actually hinder the improvement of ablation resistance. Therefore, it is required that the modified C/C composites undergo sequential oxidation over a wide temperature range while maintaining stable micro- and macrostructures in the oxide products. At this point, multicomponent UHTCs have attracted considerable interest from researchers [81,129,130].

**Figure 16:**
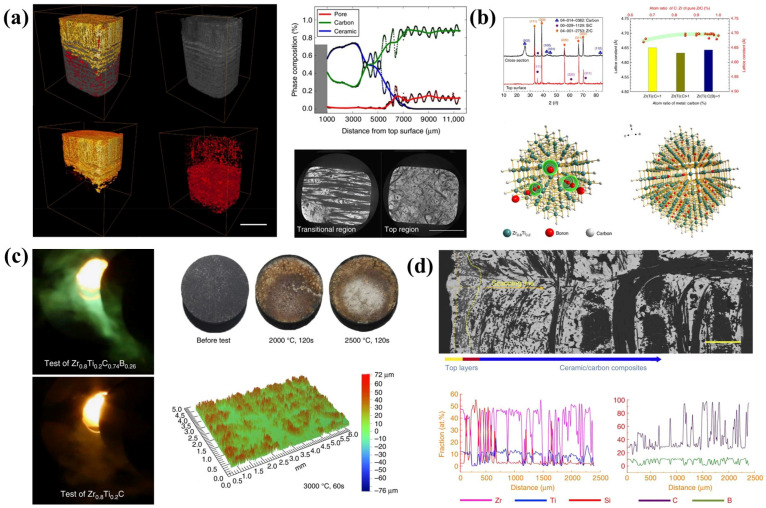
Morphology, phases composition, microstructure, and ablation performance of Zr_0.8_Ti_0.2_C_0.74_B_0.26_-modified C/C composites: (**a**) morphology of Zr_0.8_Ti_0.2_C_0.74_B_0.26_-modified C/C composites via X-ray computed tomography; (**b**) phases and structural changes in the Zr-Ti-C-B carbide during the pack cementation process; and (**c**) photograph of ablation test and morphology of tested sample. (**d**) Microstructure and element distribution of carbides within the Zr_0.8_Ti_0.2_C_0.74_B_0.26_-modified C/C composites; copied with permission from Springer Nature [23].

Regarding the multicomponent UHTCs-modified C/C composites, one of the most important works should be Zr_0.8_Ti_0.2_C_0.74_B_0.26_ modified C/C composites, which exhibit superior ablation resistance at temperatures from 2000–3000 °C, compared to existing ultrahigh-temperature ceramics (Figure 16). This carbide constitutes a Zr-Ti-based substitutional solid solution featuring carbon vacancies, which are stochastically filled by boron atoms (Figure 16b). The synergistic effects of the oxide layer’s self-sealing capability, retarded oxygen diffusion kinetics, and the presence of a densely packed, compositionally graded ceramic phase collectively contribute to a markedly reduced degradation rate of the protective oxide scale, thereby underpinning the material’s exceptional ablation resistance [23].

Long et al. investigated the growth behavior of multicomponent transition metal carbides (Hf-Zr-Ti system) during melt infiltration into porous C/C composites. The results indicate that during the initial stage of melt infiltration, a multicomponent carbide layer forms at the contact reaction interface, comprising a Zr/Ti-enriched thin layer and an Hf-enriched layer. With the process of RMI, the diffusion of Hf, Zr, Ti, and C elements promotes elemental homogenization within the multicomponent carbide layer. In addition, a transformation process in the diffusion behavior of C from sluggish diffusion to fast diffusion is discovered, which correlates with the variation in lattice mismatch caused by the diffusion of transition metal elements [105]. Gao et al. studied the effects of Hf/Zr ratios on the ablation behavior of C/C-(Hf, Zr, Ti)C composites at 2200–2500 °C using a plasma flame. The ablation rates of the composites decreased with increasing Hf/Zr ratios and increased with increasing ablation temperature. The composites with a Hf/Zr ratio of 5:3 demonstrated excellent ablation resistance, with mass and linear ablation rates of 1.14 mg/s and −1.86 μm/s at 2500 °C, respectively. The complex oxide layers synergistically enhance the ablation resistance of composites [81]. In order to improve the mechanical and ablative resistance of C/C composites, Miao et al. introduce (Hf-Ta-Zr)C single-phase solid solutions into C/C composites using a PIP method. The composites demonstrated concurrent improvements in both mechanical properties and ablation resistance. Specifically, flexural strength and modulus attained values of 219.34 MPa and 24.82 GPa. Furthermore, following 90 s of oxyacetylene torch testing, the HTZ composite exhibited low mass and linear ablation rates of 0.379 mg/s and 0.667 µm/s. This study advances the design paradigm for UHTC-modified C/C composites and extends the utility of single-phase solid solutions in ultrahigh-temperature thermal protection systems [130].

**Figure 17:**
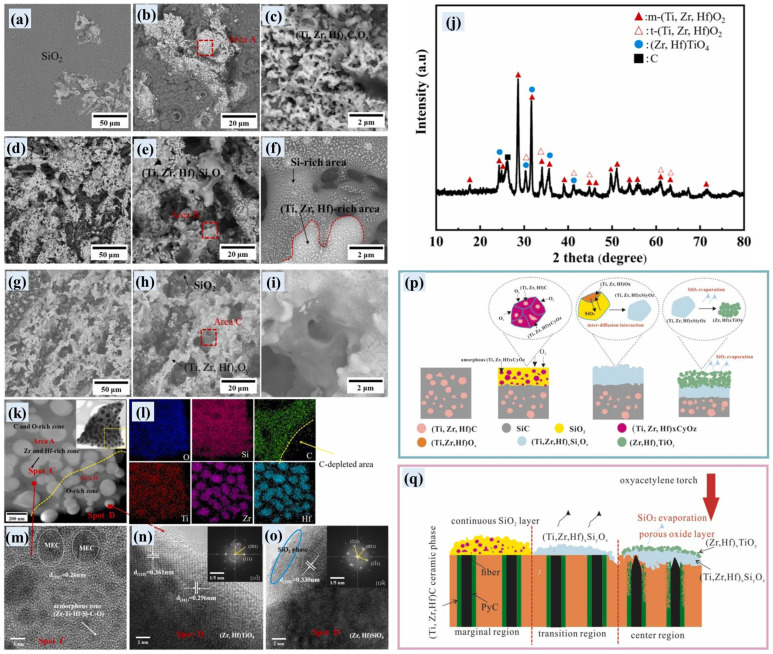
SEM images, XRD pattern, and TEM images of the oxidative products and schematic diagram of ablation mechanisms of the C/C-(Hf-Ta-Zr)C-SiC composites after 60 s ablation: (**a**–**c**) marginal region; (**d**–**f**) transitional region; (**g**–**i**) central region; (**j**) XRD pattern of the composites after ablation test; (**k**–**o**) TEM images of the oxidative product of the composites on the ablation center: (**k**) the HAADF and the (**l**) corresponding element mappings; (**m**) the HRTEM micrograph of area A; (**n**) SAED and (**o**) HRTEM micrograph in the area B; (**p**,**q**) schematic diagram of the composites during the ablation process: (**p**) evolution process of (Hf-Ta-Zr)C and its oxidation products at different areas; and (**q**) macro morphology of composites. Copied with permission from Elsevier [131].

Zhang et al. prepared C/C-(Ti, Zr, Hf)C-SiC composites with a medium-entropy carbide (Ti, Zr, Hf)C using a precursor infiltration and pyrolysis method [131,132]. The authors analyzed the surface morphologies and phase compositions of three ablation regions on the surface of the ablated composites (Figure 17a–i), which are caused by the temperature gradient and reveal the structure evolution of the ablative films during ablation. Based on the phase composition of the three ablative regions, the evolution of oxide films on the surface of the ablated composites can be summarized as follows (Figure 17p,q): Firstly, the initial oxidation of (Ti, Zr, Hf)C-SiC matrix generates (Ti, Zr, Hf)_x_C_y_O_z_ and glassy SiO_2_. Then, the inter-diffusion interaction between (Ti, Zr, Hf)O_2_ and SiO_2_ at elevated temperature generates the (Ti, Zr, Hf)_x_Si_y_O_z_ phase. With further ablation, the complete oxidation of the (Ti, Zr, Hf)_x_C_y_O_z_ and Hf-Zr-Ti-Si-C-O phases, as well as evaporation of SiO_2_, contributes to the formation of (Ti, Zr, Hf)O_2_, (Zr, Hf)TiO_4_, and (Zr, Hf)SiO_4_ phases in the ablation center.

### 3.6. High-Entropy UHTCs-Modified C/C Composites

The concept of “high entropy” originated in 2004 when Ye et al. proposed the high-entropy alloys (HEAs) alloy, which is a single-phase solid solution composed of five or more metal elements in stoichiometric or nearly stoichiometric ratios, with each element present at 5 at.%~35 at.% [133]. Due to its “high entropy” properties, it forms complex solid solutions or compounds, giving high-entropy alloys high hardness, strength, and wear resistance [134,135,136]. High-entropy ceramics (HECs) have evolved from the concept of high-entropy alloys. HECs are single-phase solid solutions formed by five or more major ceramic components [137,138], exhibiting superior oxidation resistance, creep resistance, hardness, elastic modulus, and low thermal conductivity compared to their individual components. They are a current research hotspot in the field of ceramics [5,139,140], with high-entropy ceramic carbides, nitrides, oxides, and silicides already being developed. By combining the characteristics of high-entropy with those of ceramic materials, HECs hold promise as a new generation of reliable ultrahigh-temperature materials with good toughness and erosion resistance [80,129,141].

**Figure 18:**
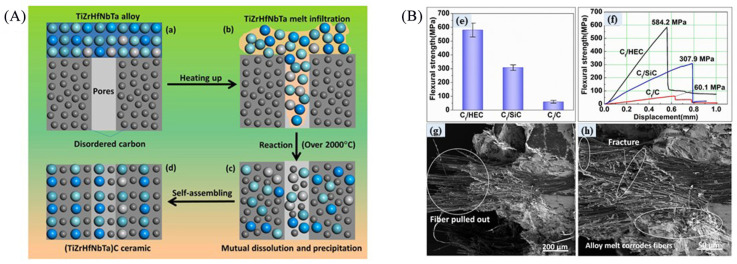
RMI process, microstructure, mechanical properties and ablation resistance of (TiZrHfNbTa)C high-entropy UHTCs-modified C/C composites (C_f_/C-(TiZrHfNbTa)C) [80]: (**A**) mechanisms and formation process of (TiZrHfNbTa)C high-entropy ceramics via TiZrHfNbTa high-entropy alloy RMI. The TiZrHfNbTa component is placed on the disordered C with voids (**a**), heated to make the mixture melt infiltrate (**b**), and then the temperature is raised to more than 2000 °C to initiate the reaction between the melt and C. the reaction products will dissolve and precipitate with each other (**c**), and finally form (TiZrHfNbTa)C ceramic (**d**); (**B**) flexural strength (**e**) and flexural strength–displacement curves (**f**) of the C/C-(TiZrHfNbTa)C and C_f_/SiC composites and C_f_/C preforms and morphologies of the fracture surfaces of the C_f_/C-(TiZrHfNbTa)C composites (**g**,**h**). Copied with permission from Elsevier [80].

He et al. [80] prepared carbon fiber-reinforced (TiZrHfNbTa)C composites (i.e., C_f_/C-(TiZrHfNbTa)C) using TiZrHfNbTa high-entropy alloy via RMI process (Figure 18A). The C_f_/C-(TiZrHfNbTa)C composites exhibit excellent mechanical properties with a bending strength of 584.2 MPa (Figure 18B). After 180 s of oxygen–acetylene ablation at 2000 °C, the linear ablation rate and mass ablation rate of the composite material were 0.6 μm/s and 0.9 mg/s, respectively. The high ablation resistance can be attributed to dense, stable, and highly viscous complex oxides (HfZrO_4_, TiNbTaO_7_, Hf_6_Ta_2_O_17_, Nb_2_Zr_6_O_17_, ZrO_2_, and HfO_2_) formed on the sample surfaces. In conjunction with the lattice distortion of (TiZrHfNbTa)C, the oxide layers effectively suppressed the invasion rate of oxygen, thus boosting the ablation resistance of the composites. Zhao et al. prepared C/C-(Ti_0.2_Zr_0.2_Hf_0.2_Nb_0.2_Ta_0.2_)C composites with the combination of the PIP and CVI process and systematically investigated their oxyacetylene ablation behavior under a heat flux of 2.4 MW/m^2^, resulting in mass and linear recession rates of 0.58 mg/s and 5.13 µm/s, respectively. After ablation, the outermost layer was completely oxidized, and a multiphase oxide layer consisting of Zr_6_Ta_2_O_17_, Hf_6_Nb_2_O_17_, Zr_6_Nb_2_O_17_, and Hf_6_Ta_2_O_17_ formed. Interestingly, their analysis revealed that the unique preferential segregation of multicomponent elements resulted in the formation of a dense interfacial transition layer composed of (Hf, Zr)TiO_4_ and (Nb, Ta, Ti)C between the oxide scale and the substrate. This layer effectively mitigated the CTE mismatch at this critical interface, facilitating the establishment of a relatively dense, Ti-enriched protective barrier that impeded subsequent oxygen ingress. That is why the C/C-(Ti_0.2_Zr_0.2_Hf_0.2_Nb_0.2_Ta_0.2_)C composites exhibit excellent oxyacetylene ablation behavior under this ultrahigh-temperature condition [142].

**Figure 19:**
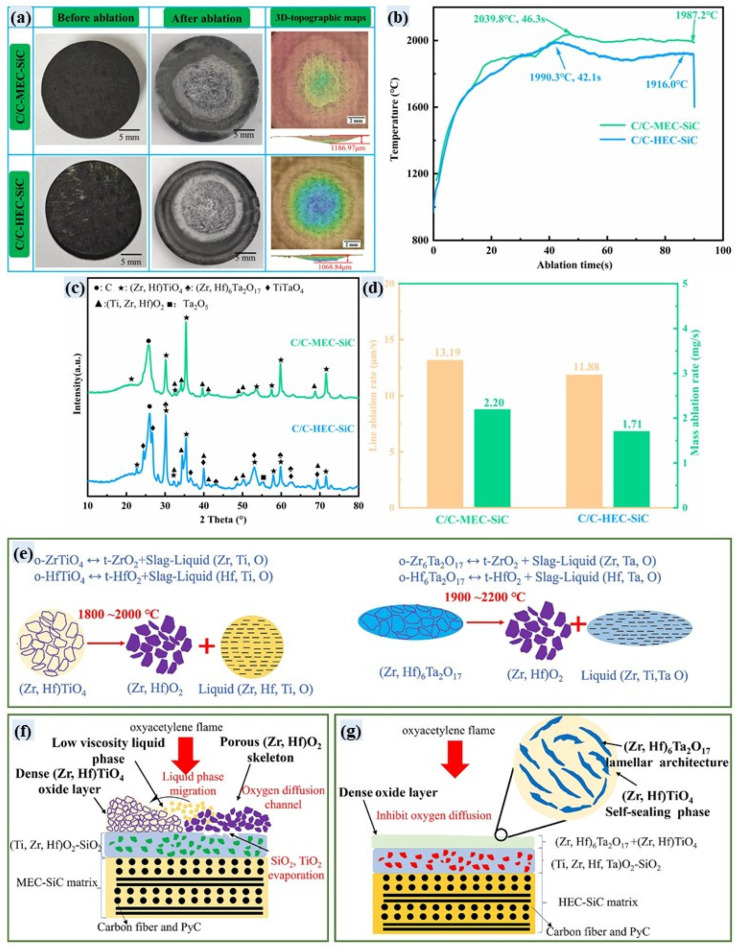
(**a**) Typical macrophotographs of the C/C-MEC-SiC and C/C-HEC-SiC before and after ablation test (MEC and HEC represent the (Ti, Zr, Hf)C and (Ti, Zr, Hf, Ta)C, respectively) and 3D morphology of the samples after ablation; (**b**) surface temperature curves during the ablation; (**c**) XRD patterns of the ablated samples; (**d**) mass and linear ablation rate of the two samples; (**e**) the mechanisms of reversible peritectic transition on the surface of the sample during ablation; and the ablation mechanisms at central region of the (**f**) C/C-MEC-SiC and (**g**) C/C-HEC-SiC, respectively. Copied with permission from Elsevier [132].

Zhang et al. [132] systematically compared the ablation resistance of the high-entropy UHTCs-modified C/C composites (i.e., C/C-(Ti, Zr, Hf, Ta)C-SiC) with the medium-entropy UHTCs-modified C/C composites (i.e., C/C-(Ti, Zr, Hf)C-SiC), which are prepared using the same PIP process. The results show that the C/C-(Ti, Zr, Hf, Ta)C-SiC composites exhibit much better ablation resistance than that of the C/C-(Ti, Zr, Hf)C-SiC composites under a heat flux of 3.2 MW/m^2^ (Figure 19a–d). The enhanced ablation resistance observed in C/C-(Ti, Zr, Hf, Ta)C-SiC composites is attributed to the development of a Ta-doped oxide film combined with the high reversible peritectic temperature characteristic of (Hf, Zr)_6_Ta_2_O_17_. Figure 19e–g summarize the ablation mechanisms of the two composites. During ablation, the (Zr, Hf)TiO_4_ phase in C/C-(Ti, Zr, Hf)C-SiC composites experiences a reverse peritectic reaction, decomposing into a low-viscosity liquid TiO_2_ oxide alongside a porous (Zr, Hf)O_2_ particle skeleton. This low-viscosity liquid phase is readily scoured away from the ablation zone, leaving behind a porous t-(Zr, Hf)O_2_ skeleton that inadequately blocks oxygen ingress (Figure 19e,f). Consequently, the composite exhibits poor ablation resistance. Introducing Ta into the C/C-(Ti, Zr, Hf, Ta)C-SiC composite system enhances the stability of the resulting multiphase oxide film and reduces its overall volatility. Owing to the lowest volatilization pressure of Ta_2_O_5_, it can effectively fill the defects formed by the volatilization of Si and Ti oxides and greatly inhibits further oxygen erosion of the substrate. Furthermore, the high reversible peritectic temperature of (Hf, Zr)_6_Ta_2_O_17_ minimizes the generation of low-viscosity liquid phases during ablation. This reduction in fluid phases mitigates material loss due to mechanical erosion (Figure 19e,g). Therefore, the low volatility and high stability of oxide film are the key to improve the ablation property of UHTCs-modified C/C composites [132].

## 4. Summary and Outlook

UHTCs-modified C/C composites have become promising thermal structures and protective materials for engine propulsors and hypersonic vehicles due to their excellent oxidation resistance, high strength, high fracture toughness, good fault tolerance as well as excellent thermal shock resistance. The content and contribution of this work can be summarized as follows:

(1) Systematic compilation and comparison: This work comprehensively compiles and critically compares the fabrication strategies, ablation behaviors, and protective mechanisms across diverse UHTC modification systems, including single-phase (e.g., ZrC and HfC), multiphase (e.g., C/C-ZrC-SiC), multicomponent (e.g., Zr-Ti-C-B solid solutions), and the emerging high-entropy ceramics (e.g., (TiZrHfNbTa)C). This comparison elucidates performance differences and synergistic effects, providing a clear roadmap for material design.
(2) In-depth mechanistic insights: This review offers a profound analysis of the failure mechanisms of C/C-UHTCs composites under extreme thermo-mechano-chemical coupled environments. Crucially, it deciphers the formation, evolution, and failure modes of oxide layers (e.g., ZrO_2_ and HfO_2_) and highlights the pivotal role of introducing secondary phases (e.g., SiC) or forming multicomponent/high-entropy oxides (e.g., complex silicates and high-entropy oxides) in improving oxide layer density, stability, self-healing capability, and thermal compatibility. This deepens the fundamental understanding of the protection mechanisms at ultrahigh temperatures.
(3) Critical evaluation of innovative processes: It critically assesses the advantages and limitations of key fabrication techniques—CVI, PIP, RMI, and their hybrids (e.g., SI + PIP and molten salt-assisted RMI)—in controlling ceramic phase content, distribution, interface structure, and ultimately composite performance. The review underscores the potential of process innovations (e.g., molten salt assistance and vacuum vibration-assisted slurry infiltration) in overcoming drawbacks of traditional methods (low efficiency, fiber damage, and metal residue), pointing towards pathways for efficient fabrication of high-performance composites.
(4) Clarification of challenges and future directions: It explicitly identifies the core challenges hindering the practical applications of C/C-UHTCs composites (e.g., precise control of preform pore structure, fabrication of large/complex-shaped components, thorough understanding of ablation mechanisms in multicomponent/high-entropy systems, cost reduction, bridging ground–space simulation gaps). Building on current knowledge, it outlines critical future research priorities, encompassing advanced structural design (e.g., graded structures and nanowire reinforcement), development and application of novel high-entropy/multicomponent ceramics, integration of multi-scale modeling with real-service environment testing, and breakthroughs in low-cost, scalable manufacturing technologies.

This review not only serves as a comprehensive knowledge base for researchers to understand the materials science fundamentals and performance optimization strategies of C/C-UHTCs, but more significantly, it establishes a solid theoretical foundation for designing next-generation high-performance, long-lifetime thermal protection materials for extreme environments (e.g., hypersonic vehicle leading edges and engine hot-section components). It also clearly delineates the critical research pathways essential for advancing the field. Future breakthroughs will heavily rely on the deep integration of material design, processing technology, characterization methods, and theoretical modeling to ultimately realize the scalable and highly reliable application of C/C-UHTC composites in the aerospace sector.

Nevertheless, it is worth noting that UHTCs-modified C/C composites still face many challenges: (1) the design and control of the pore structure of the starting C/C composites should be further investigated in order to more efficiently control the content and distribution of the introduced UHTCs; (2) the technology for preparing large and irregular components needs improvement to meet the requirements of practical applications; (3) the oxidation/ablation mechanisms of multiphase/component UHTCs should be further clarified to support the design of high-performance UHTCs-modified C/C composites; (4) the cost of these composites should be strongly reduced for large scale applications; and (5) the testing methods and simulation technologies should be optimized to more accurately simulate the real-world service conditions and reduce the differences between the ground and space. These issues will constrain the applications of UHTCs-modified C/C composites in actual spacecraft equipment. Therefore, preparing high-performance UHTCs-modified C/C composites and achieving efficient, low-cost manufacturing remain long-term goals.

## Figures and Tables

**Table 1:** Physical and mechanical properties of boride, carbide, and nitride-based UHTCs. Copied with permission from Springer Nature [5].

Material	Crystal Structure	Melting Point (°C)	Density (g/cm^3^)	CTE, α (10^−6^ K^−1^)	Thermal Conductivity (W·m^−1^·K^−1^)	Electrical Resistivity (μΩ·cm)	Elastic Modulus (GPa)		Hardness (GPa)
							Cal.	Exp.	
Boride									
TaB_2_	HCP	3040	12.5	8.2–8.8	10.9–16.0	33	497	551	19.6
TiB_2_	HCP	3225	4.5	7.6–8.6	64.4	16–28.4	583	575	24.0
ZrB_2_	HCP	3245	6.1	5.5–8.3	57.9	9.2	523	489	23.0
HfB_2_	HCP	3380	11.2	6.3–7.6	51.6	8.8–11	535	451	28.0
Carbide									
TiC	FCC	3100	4.9	7.5–7.7	17–21	52.5	455	437	30.0
ZrC	FCC	3530	6.6	6.82	20.61	68.0	436	387	25.0
TaC	FCC	3800	14.5	6.6–8.4	22.2	30–42.1	550	537	17.0
HfC	FCC	3900	12.8	6.3	22.2	45.0	537	461	24.2
Nitride									
TaN	FCC	2900	13.4	3.2	8.3	128–135	490	490	10.8
TiN	FCC	2950	5.4	9.35	29.1	21.7	463	400	18.6
ZrN	FCC	2950	7.3	7.24	20.9	13.6	390	384	15
HfN	FCC	3385	13.9	6.5	21.6	33	411	398	16.1

**Table 2:** Process and ablation performance of UHTCs-modified C/C composites.

Materials	Method	Ablation Method	Time (s)	Heat Flow (MW/m^2^)	Linear Ablation Rate (μm/s)	Mass Ablation Rate (mg/s/cm^2^)	Ref.
C/C-ZrC	SI + TCVI	Oxygen–acetylene	60	4.200	0.510	0.620	[65]
C/C-ZrC	ICVI + RMI	Oxygen–acetylene	60	2.380	0.264	0.422	[69]
C/C-ZrC	PIP	Oxygen–acetylene	120	2.380	−1.230	−1.640	[70]
C/C-ZrC-SiC	RMI	Oxygen–acetylene	120	2.380	−0.028	0.545	[71]
C/C-ZrC-SiC	RMI	Oxygen–acetylene	300	-	−0.280	0.870	[72]
C/C-ZrB_2_-SiC	CVI + PIP	Oxygen–acetylene	120	2.400	2.450	0.0095	[31]
C/C-HfC	PIP	Air plasma	240	-	5.310	0.550	[73]
C/C-HfC	SI	Oxygen–acetylene	60	-	0.720	0.12	[74]
C/C-HfC_nw_	PIP + ICVI	Oxygen–acetylene	20	-	7.500	3.200	[75]
C/C-HfB_2_	PIP + CVI	Oxygen–acetylene	90	4.180	6.560	0.275	[76]
C/C-ZrC-SiC	RMI	Oxygen–acetylene	120	3.860	0.415	0.500	[44]
C/C-ZrB_2_-ZrC-SiC	PPI	Air plasma	180	-	2.610	5.09	[77]
C/C-SiC-ZrC-HfC	RMI	Oxygen–acetylene	120	-	−1.100	1.500	[78]
C/C-SiC-ZrC-HfC	PIP	Oxygen–acetylene	120	2.380	0.255	0.151	[79]
C/C-Zr_0.8_Ti_0.2_C_0.74_B_0.26_-SiC	RMI	Oxygen–acetylene	60	-	−0.3	0.01	[23]
C/C-ZiC-ZrC-SiC	CVI + PIP + RMI	Air plasma	120	-	11.00	4.17	[14]
C_f_/(TiZrHfNbTa)C	RMI	Oxygen–acetylene	180	-	0.600	0.900	[80]
C/C-(Hf_0.5_, Zr_0.3_, Ti_0.2_)C	RMI	Air plasma	240	5.520	−1.860	1.14	[81]
C/C-(Hf_0_._5_Zr_0_._5_)C	PIP	Air plasma	60	2.300	3.15	−0.25	[82]
C/C-ZrC-SiC	CVI + PIP	Air plasma	180	-	0.194	1.73	[83]

## Data Availability

No new data were created or analyzed in this study.
